# Comprehensive analysis of DNA polymerase III α subunits and their homologs in bacterial genomes

**DOI:** 10.1093/nar/gkt900

**Published:** 2013-10-06

**Authors:** Kęstutis Timinskas, Monika Balvočiūtė, Albertas Timinskas, Česlovas Venclovas

**Affiliations:** Institute of Biotechnology, Vilnius University, Graičiūno 8, Vilnius LT-02241, Lithuania

## Abstract

The analysis of ∼2000 bacterial genomes revealed that they all, without a single exception, encode one or more DNA polymerase III α-subunit (PolIIIα) homologs. Classified into C-family of DNA polymerases they come in two major forms, PolC and DnaE, related by ancient duplication. While PolC represents an evolutionary compact group, DnaE can be further subdivided into at least three groups (DnaE1-3). We performed an extensive analysis of various sequence, structure and surface properties of all four polymerase groups. Our analysis suggests a specific evolutionary pathway leading to PolC and DnaE from the last common ancestor and reveals important differences between extant polymerase groups. Among them, DnaE1 and PolC show the highest conservation of the analyzed properties. DnaE3 polymerases apparently represent an ‘impaired’ version of DnaE1. Nonessential DnaE2 polymerases, typical for oxygen-using bacteria with large GC-rich genomes, have a number of features in common with DnaE3 polymerases. The analysis of polymerase distribution in genomes revealed three major combinations: DnaE1 either alone or accompanied by one or more DnaE2s, PolC + DnaE3 and PolC + DnaE1. The first two combinations are present in *Escherichia coli* and *Bacillus subtilis*, respectively. The third one (PolC + DnaE1), found in *Clostridia*, represents a novel, so far experimentally uncharacterized, set.

## INTRODUCTION

DNA polymerase III is a tripartite protein machine responsible for replication of bacterial genome (1–5). It consists of a DNA polymerase, its processivity factor β-clamp and a clamp loader complex. The actual DNA synthesis is performed by the polymerase III α-subunit (PolIIIα), classified into the C-family of DNA polymerases (6). Surprisingly, bacterial PolIIIα subunits are both structurally and evolutionary distinct from eukaryotic and archaeal replicative DNA polymerases (7,8) that belong to the B-family. Instead, the PolIIIα catalytic domain is distantly related to the X-family of DNA polymerases (7,8), exemplified by eukaryotic Polβ, a polymerase acting in DNA excision repair (9,10). It should be noted that this unexpected relationship could not be detected by protein sequence comparison and only became apparent in the context of 3D structures (7,8). Although polymerases of C and X families are not globally similar, a strong case for their common evolutionary origin could be made based on the observation that they share a common fold of corresponding ‘palm’ domains and bind DNA in the same manner (11). In contrast, ‘palm’ domains of DNA polymerases belonging to A, B and Y families have entirely different fold. Taken together, these findings lend additional support for the hypothesis that bacterial replicative polymerases (C-family) on one hand and archaeal/eukaryotic replicative polymerases (B-family) on the other hand have evolved as components of two independent DNA replication systems (12). Another interesting observation is that C-family polymerases are essentially confined to the bacteria kingdom. Only a handful of PolIIIα homologs have been detected in bacteriophages, which predominantly use B-family (and to lesser extent A-family) DNA polymerases (13,14). One of the explanations for the scarcity of PolIIIα homologs even in bacteria-infecting viruses is that the C-family is evolutionary ‘young’ compared with the B-family (13). Owing to their relatively late emergence, C-family DNA polymerases might have failed to make a significant imprint in the B-family–dominated viral landscape (13), and a few instances of C-family members in bacteriophages might be the result of lateral gene transfer events from bacteria (15).

**Figure 1.**
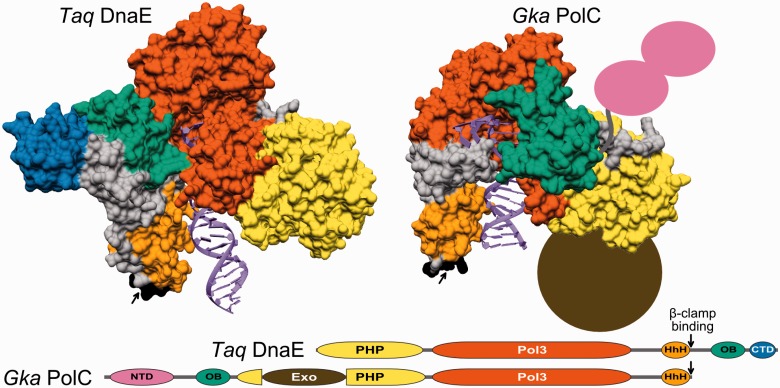
Structural organization of DnaE and PolC forms of C-family DNA polymerases. Crystal structures of *T. aquaticus* DnaE (left, PDB ID: 3E0D) and *G. kaustophilus* PolC (right, PDB ID: 3F2B) complexes with the DNA displayed in same orientation. Protein structures are shown as solvent accessible surfaces with different structural modules shown in different colors. The missing NTD and the exonuclease domain (Exo) in PolC structure are represented correspondingly by a pair of ellipses (purple) and a filled circle (brown). Linear domain organization for both polymerase forms is indicated at the bottom. Domain labels: PHP, the polymerase and histidinol phosphatase domain; Pol3, the polymerase core consisting of ‘palm’, ‘thumb’ and ‘fingers’; HhH, the tandem helix–hairpin–helix motif; OB, oligonucleotide binding domain; CTD, C-terminal domain; NTD, N-terminal domain consisting of two subdomains; Exo, an integral proofreading 3′–5′ exonuclease domain. Colors for individual domains correspond to those in structural representation. The β-clamp binding motifs are indicated by black arrows.

PolIIIα subunits come in two major forms, DnaE (7,8) and PolC (16). A typical example of DnaE is PolIIIα of the extensively studied model organism, Gram-negative bacterium *Escherichia coli*. PolC is present in low-GC Gram-positive bacteria such as *Bacillus subtilis*. The two different PolIIIα forms are thought to be the result of an ancient gene duplication event predating the radiation of Gram-positive and Gram-negative bacteria (17). The two forms undoubtedly share common evolutionary origin, yet they differ by the exact composition and the arrangement of structural domains (7,8,16). Both have the polymerase and histidinol phosphatase (PHP) domain, the polymerase core consisting of ‘palm’, ‘thumb’ and ‘fingers’, and the tandem helix–hairpin–helix (HhH)_2_ motif followed by the β-clamp binding motif, all arranged in the same order (Figure 1). When it comes to differences, the oligonucleotide binding (OB) domain present in both forms is embedded in the opposite regions of the polypeptide chain. In DnaE-type PolIIIα, it is C-terminal to the β-clamp binding motif, while in PolC, it is N-terminal to the PHP domain. In addition, DnaE and PolC possess structural domains unique to each form. DnaE has a small structural domain at the extreme C-terminus. In contrast, PolC has an unrelated N-terminal domain (NTD) predicted to have two type II KH-like subdomains (18). Moreover, PolC has an integral proofreading 3′–5′ exonuclease domain inserted into the PHP domain, while the DnaE proofreading exonuclease activity is provided by ε, a separate subunit.

If the number of distinct PolIIIα subunits and their role in a bacterial cell are considered, there also are notable differences. The widely studied *E. coli* encodes a sole DnaE-type PolIIIα subunit, which performs DNA synthesis of both leading and lagging strands (1,19,20). However, this is not a universal situation in the bacterial world. For example, low-GC Gram-positive bacteria were found to have both PolC and DnaE (17). Experiments with *B. subtilis* and some other Gram-positive bacteria showed that both types of PolIIIα subunits are essential (21–23). Initially, it was thought that PolC and DnaE are leading and lagging strand polymerases, respectively (21). However, more recently, *in vitro* experiments with the reconstituted *B. subtilis* replisome (24) revealed a different picture of their division of labor. It turned out that DnaE makes an initial extension of the RNA primer on both strands and then PolC takes over for rapid synthesis of long stretches of DNA (24). In this regard, *B. subtilis* DnaE is reminiscent of eukaryotic Pol α, which extends the RNA primer and then makes way for a processive replicase (25). Some bacteria have a second copy of DnaE, usually referred to as DnaE2. So far, genetic studies targeting *dnaE2*, all without a single exception, identified it as a nonessential gene (26–32), indicating that DnaE2 is not required for chromosomal DNA replication. Instead, DnaE2 has been associated with DNA damage-inducible error-prone translesion DNA synthesis (TLS) (26–28,31,32). In genomes, *dnaE2* is typically found as part of LexA-regulated contiguous or split multigene cassette, which includes two other genes, *imuA/imuA’* and *imuB* (27,33,34). The two genes encode catalytically inactive homologs of RecA and Y-family DNA polymerase, respectively (29,35). In the most detailed study to date, it was shown that in *Mycobacterium tuberculosis*, both genes along with *dnaE2* are necessary for induced mutagenesis, but the error-prone TLS is directly linked to DnaE2 (35). Nonetheless, the role of DnaE2 as an error-prone TLS polymerase might not be general. For example, the *dnaE2* disruption in two *Pseudomonas* species (*Pseudomonas aeruginosa* and *Pseudomonas putida*) produces opposite effects (28,29). Moreover, a recent study was unable to associate any phenotype with the *Streptomyces coelicolor dnaE2* mutant (30). *Streptomyces coelicolor dnaE2* was found to be SOS-inducible, but it was dispensable for DNA replication, linear chromosome end patching, ultraviolet resistance or mutagenesis. Whether the observed *dnaE2* phenotypic differences reflect intrinsic DnaE2 properties or the differentially controlled access of DnaE2 to the sites of DNA synthesis remains unclear.

Except for a handful of bacterial species, C-family DNA polymerases have not been studied in detail experimentally. On the other hand, the increasing availability of bacterial genome sequences provides a possibility to explore the diversity and distribution of PolIIIα subunits in bacteria using computational methods. An earlier survey of annotated PolIIIα subunits within 159 fully sequenced bacterial genomes (36) partitioned C-family DNA polymerases into four major groups, namely, PolC and three DnaE groups (DnaE1, DnaE2 and DnaE3). The survey has also found that different types of PolIIIα subunits have different preferred combinations within bacterial genomes suggesting different degree of versatility and mutual compatibility for individual groups of C-family polymerases (36).

In this study, we performed a comprehensive analysis of C-family DNA polymerases (putative PolIIIα subunits and their homologs) identified in a much larger sample (close to 2000) of complete bacterial genomes. We took advantage of the available 3D structures of PolIIIα representatives and surveyed various sequence, structure and surface properties as well as their differences within and between distinct groups of C-family polymerases. Among other things, the results enabled us to suggest a specific evolutionary pathway leading to the emergence of DnaE and PolC from the common ancestor. We also surveyed the combinations of PolIIIα homologs found in genomes in an attempt to get more clues about functional properties of different polymerase groups and a deeper insight into the evolution of bacterial replication systems. As a result, in addition to two typical replication systems represented correspondingly by *E. coli* and *B. subtilis*, our analyses suggested the existence of a third, so far uncharacterized, replication system in *Clostridia*.

## MATERIALS AND METHODS

### Bacterial genomes and protein sequence data

Annotated complete bacterial genomes (Supplementary Table S1) were obtained from NCBI (ftp://ftp.ncbi.nlm.nih.gov/genomes/Bacteria/). The associated data on bacterial taxonomic classification, various physiological properties, metabolic features and habitats were obtained from the Integrated Microbial Genomes database (http://img.jgi.doe.gov/). C-family DNA polymerases (DNA PolIIIαs and their homologs) were identified by performing protein sequence searches with PSI-BLAST (37) against the protein database derived from the collected bacterial genomes. PSI-BLAST searches were run until convergence (E-value = 1e–03 inclusion threshold) using conserved polymerase regions (corresponding to the 324–788 region of the *E. coli* PolIII α-subunit) of C-family representatives as search probes. To make sure that no unannotated sequences were missed, representatives of C-family were also used to search the collection of genomic sequences using TBLASTN (E-value = 1e–03 significance threshold). Results of both types of searches were combined. If a polymerase sequence contained an intein, it was excised before further analysis. A number of cyanobacterial polymerase sequences are split by an intein (38). Such sequences were joined before the intein removal. A small number of sequences were found fragmented, usually due to frameshifts that at least in some cases could be the result of sequencing/assembling errors. Therefore, if closely related sequences were found intact in other genomes, the fragmented sequences were also reconstructed to avoid false-negative results in polymerase distribution studies. The N-termini of some sequences, missing as a result of wrong selection of the translation start site, were corrected based on comparison with close homologs. Any remaining sequence fragments without an intact polymerase active site were removed from further analysis.

A nonredundant set of genomes was constructed as follows. If any two genomes encoded the same number of C-family polymerases and corresponding polymerase sequences in both of them were >90% identical [determined by clustering with CD-HIT (39)], only one of the two genomes was included in the nonredundant set. If the number of C-family polymerases encoded in the two genomes was different, both genomes were included independently of sequence similarity. As a result, groups of closely related genomes (typically different strains of the same species) were represented by a single genome, significantly reducing the redundancy of genomic data.

### Multiple sequence alignments and the analysis of sequence features

Multiple sequence alignments in all analyses were constructed with MAFFT (40) using the accuracy-oriented mode (L-INS-i). Predictions of protein secondary structure and disordered regions were carried out using, respectively, PSIPRED (41) and DISOPRED (42). Theoretical isoelectric points (pIs) for protein sequences were calculated using the ‘Compute pI/Mw’ tool on the ExPASy server (43).

### Phylogenetic analysis

Initially, full-length sequences of the nonredundant set were aligned. The highly conserved PHP-Pol3-(HhH)_2_ region was then excised and the corresponding sequence regions were realigned. The alignment was then reduced by removing positions with at least 50% gaps to increase the signal-to-noise ratio of the subsequent phylogenetic analysis. A maximum-likelihood phylogenetic tree was constructed using RAxML program (44). The tree was constructed using Le and Gascuel model of amino acid substitution (45) with the use of the Γ model of rate heterogeneity. This model was selected as best fitting the analyzed sequences using the program ProtTest (46), run with standard parameters. The best tree was selected from 160 distinct tree inferences. The reliability of subtrees was inferred using the widely accepted bootstrap method (47) as implemented in RAxML. The bootstrap support values were obtained after 1000 generations. Tree analysis and visualization were carried out using Dendroscope (48) and iTOL (49).

### Identification of conserved structural domains

Conserved structural-functional domains in polymerase sequences were identified using three different approaches designed to maximize the sensitivity of domain detection.

First, *E. coli* DnaE and *B. subtilis* PolC sequence regions corresponding to structural-functional domains defined based on DnaE and PolC crystal structures (Figure 1) were used as queries for PSI-BLAST searches (up to five iterations; the inclusion threshold E-value = 1e–02) against polymerase sequences. Significant sequence matches (E-value ≤ 1e–03, at least 40% of query sequence aligned, 10% or higher sequence identity) were retained.

Next, for each of the PolIIIα domains, the sequences identified in the PSI-BLAST searches and filtered to 90% sequence identity were used to construct domain-specific Hidden Markov Models (HMMs). HMMs constructed from MAFFT alignments using the HMMer software suite (50) were appended to the full HMM collection of PFAM (51) domains. Every polymerase sequence was then scanned against the ‘extended’ PFAM database with *hmmscan* (HMMer suite). Matches with E-value ≤ 1e–03 were considered significant.

The two above approaches were sufficiently sensitive to identify Pol3, PHP, (HhH)_2_ and Exo domains. At the same time, for a number of polymerase sequences, no conserved domains with statistically significant values were identified in the N- and C-terminal regions, where at least OB domain and CTD (in the case of DnaE) or NTD and OB domain (for PolC) could be expected. To further test the domain composition of these terminal regions, HHsearch (52), a more sensitive homology detection method based on the HMM-HMM comparison, was used. To this end, domain-specific HMMs were constructed from the updated multiple sequence alignments for NTD, CTD and OB domains using the HHsearch suite instead of HMMer. The resulting HMMs were then appended to the HHsearch-specific PFAM domain database (53). For every query, an HMM was constructed using three iterations (E-value = 1e–03) of either HHblits (54) against nr20 (the NCBI nonredundant protein sequence database, filtered to the maximum of 20% sequence identity) or PSI-BLAST against nr80. Query HMM was then used to search the ‘extended’ PFAM domain HMM database. Iterative methods (e.g. PSI-BLAST) often tend to overextend alignments into neighboring nonhomologous domains producing false-positive matches (55). To avoid including more than a single domain into the query alignment and subsequently into the corresponding query HMM, the following procedure was used. For unassigned sequence regions, a short fragment of terminal 50 residues was initially used as a query. If none of the conserved domains were detected, additional searches were performed by gradually extending the query region. After each extension, the results were inspected for the presence of false positives (already assigned neighboring domains, e.g. OB domain). The domain assignment was considered reliable (true positive) if its HHsearch probability was ≥90% for this particular domain and at the same time at most 30% probability for unrelated domains.

### Analysis of functional motifs

For full-length sequences of each polymerase group, separate multiple sequence alignments were generated. The alignments were inspected visually and adjusted manually if necessary. For each analyzed motif, relative positions from multiple sequence alignments for each group were extracted. In cases where multiple nonconsecutive positions were analyzed (e.g. the metal binding site of the PHP domain), concatenated alignments for each group were made. The WebLogo (56) representation of resulting alignments was used to visualize the distribution of residues (or deletions, represented by gray squares) in each position.

### Analysis of residue conservation and 3D structure surface properties

All analyzed polymerases with no known 3D structure were modeled using homology modeling approach. Structures of *Thermus aquaticus* DnaE1 (PDB ID: 3E0D) and *Geobacillus kaustophilus* PolC (PDB ID: 3F2B) with bound DNA were used as templates to generate models for DnaE and PolC polymerases accordingly. Sequence-structure alignments were generated using HHsearch. The 3D structural models were constructed with Modeller (57) and then evaluated using Prosa2003 (58) and visual inspection. In the case of visible flaws, models were iteratively refined (59). The analysis of surface residue conservation was performed using the ConSurf (60) server supplied with locally constructed multiple sequence alignments for each of the analyzed groups. Surface electrostatic properties of all structures were computed using the APBS server (61). Before electrostatics calculation, the structures were prepared using the PDB2PQR server (62) with the PARSE force field. Visualization and analysis of 3D structures was performed with UCSF Chimera (63).

## RESULTS

Initially, we identified all putative C-family DNA polymerases (PolIIIα subunits and their homologs) in 1877 completely sequenced bacterial genomes. Once the polymerases (2956 in total) were compiled, we selected a representative nonredundant set of bacterial genomes as described in ‘Materials and Methods’ section. This mostly removed closely related strains of the same species that otherwise might have strongly biased the data. All the subsequent analyses were performed using the representative set of 945 bacterial genomes coding for 1590 putative C-family DNA polymerases (the redundancy of both genomes and polymerases was reduced approximately twice). Detailed information about each of the representative polymerases is presented in Supplementary Table S1.

### Distinct polymerase groups

**Figure 2.**
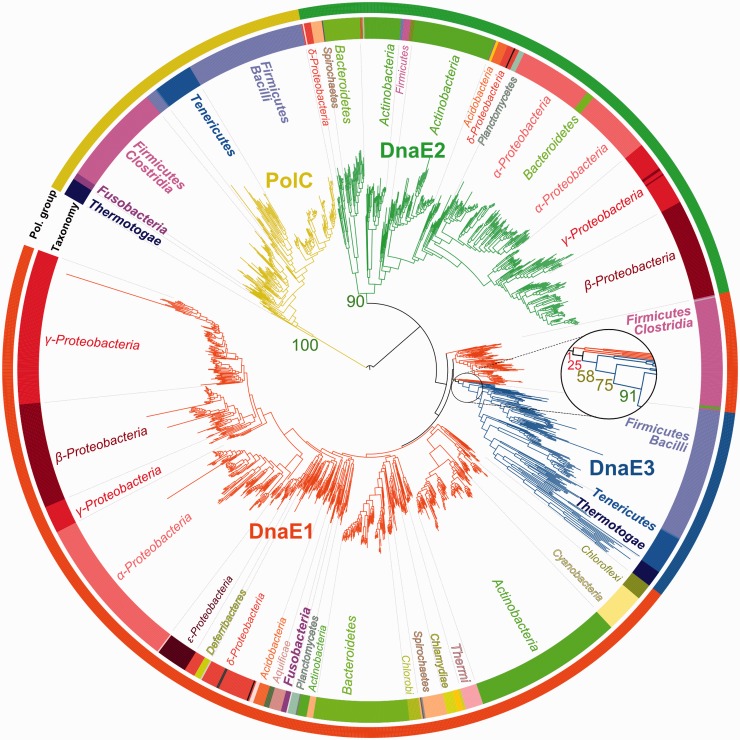
Phylogenetic tree of C-family polymerases. Tree colors correspond to four distinct polymerase groups (labeled). The tree is arbitrarily rooted at PolC. Bootstrap values, relevant to polymerase group separation, are shown (numbers). The zoomed area shows the DnaE3 branching area. Bacterial taxonomy is represented by both a colored strip (inner) and text. Minor (less than five bacteria in the set) taxonomic groups are shown only in colors. PolC-containing taxa are shown in bold. For clarity, *Clostridia* and *Negativicutes* classes (taxonomically closely related) of phylum *Firmicutes* are merged.

The distinction between the two major forms (DnaE and PolC) of C-family polymerases has been noticed some time ago (17). We used phylogenetic analysis to reveal evolutionary partitioning of the C-family at a higher resolution. Based on the available crystal structures of DnaE (7,8) and PolC (16) representatives, we first defined a conserved region that is shared by both forms (Figure 1) and includes PHP, Pol3 and (HhH)_2_ domains. We then used this region to analyze the evolutionary relationship between C-family polymerases. Phylogenetic analysis revealed that the two major forms are well-separated (100% bootstrap support) and yet show a different degree of diversity (Figure 2). PolC polymerases, typified by one of the two essential *B. subtilis* PolIIIα subunits (PolC), represent a single, evolutionary compact, group. In contrast, DnaE-type polymerases are significantly more diverse. The most distinct among DnaE-type polymerases is the DnaE2 group (90% bootstrap support). One of the best characterized members of this group is *M. tuberculosis* DnaE2, a nonessential error-prone DNA polymerase (26,35). Overall, the remaining phylogenetic tree is poorly resolved. Nevertheless, a group that includes *B. subtilis* DnaE, the second essential PolIIIα subunit in addition to PolC, stands out. Following the previously introduced nomenclature (36), we labeled this group as DnaE3. The consideration of DnaE3 polymerases as a distinct group is supported by the high bootstrap value (91%). However, there is unavoidable ambiguity in putting the exact separation line between the DnaE3 group and the remaining sequences. Therefore, we chose to assign all the sequences that clustered together more often than not (bootstrap support >50%, see the zoomed section in Figure 2) to the DnaE3 group. The remaining sequences were assigned to the DnaE1 group. A well-characterized representative of the DnaE1 group is the *E. coli* PolIIIα subunit, which is the sole high fidelity replicative C-family DNA polymerase in the cell. Notably, DnaE1 is a large and diverse group. Therefore, it might be argued that DnaE3 may be considered as just one of many subgroups. However, the separation of the DnaE3 group is also supported by the analysis of a number of different features presented in the sections below.

To maximize phylogenetic tree resolution, a minimal number of strongly diverged sequences were removed from the analysis. All of the omitted sequences, except three, were present as additional copies in respective genomes, suggesting that they are not the primary replicative polymerases. They were labeled DnaEX, while the three essential polymerases were assigned to the specific polymerase group based on the sequence similarity alone.

### Genomic distribution

Having defined distinct polymerase groups, we next analyzed which polymerase combinations are observed in individual genomes. Are there preferred, avoided or even incompatible (not observed) combinations? Based on the results of the analysis presented in Table 1, several observations can be made. Importantly, we did not find a single genome that would entirely lack C-family polymerases. This finding implies that bacteria universally use C-family polymerases for the replication of their genome, and that there are no alternative nonhomologous functional solutions. The majority of bacteria have two, three or even four putative C-family polymerases. However, a large fraction (41%) of genomes encode a single polymerase of the DnaE1 type (like in *E. coli*). Consistent with an earlier study (36), we find that DnaE1 is not only the most widely distributed, but also the only one that may exist in a genome either alone or in combination with polymerases from other groups. Members of other groups (PolC, DnaE3 and DnaE2) are always accompanied by at least one representative of a different group. PolC always co-occurs with either DnaE3 or DnaE1. Unlike widely distributed DnaE1, PolC is confined to several bacterial phyla, namely *Firmicutes*, *Fusobacteria*, *Tenericutes* and *Thermotogae* (Figure 2 and Table 1). These mostly include Gram-positive bacteria with low genomic GC content. In particular, an interesting situation is observed in *Firmicutes*. In these bacteria, PolC is accompanied by DnaE-type polymerases, which, according to phylogenetic analysis, are split into DnaE1 and DnaE3. The split essentially coincides with the taxonomic division as DnaE3 polymerases are found in class *Bacilli*, while DnaE1 are found in classes *Clostridia* and *Negativicutes*. *Fusobacteria*, represented by only five genomes in our set, is the only other phylum in which PolC was found in combination with DnaE1 sequences. DnaE3 always co-occurs with PolC, while DnaE2 polymerases almost exclusively go together with DnaE1. DnaE2s appear to be distributed randomly, and even related bacterial species may differ by the presence (absence) of DnaE2. Although in general C-family polymerases are rarely encoded in plasmids (only 3% in our set), of those that are, about two-thirds are DnaE2s. This observation suggests that horizontal gene transfer may be an important route of the DnaE2 dispersal within bacterial genomes. However, despite the inferred ‘mobility’ of DnaE2 polymerases, they are extremely rarely found in PolC genomes (Table 1). This may be interpreted as the conflicting overlap between the function of DnaE2 and that of a PolC-DnaE3/DnaE1 pair.

**Table 1.** Combinations of C-family DNA polymerases in 945 bacterial genomes

Genome count	Fraction of genomes (%)	Number of polymerases	Polymerase combination	Taxonomic spread
**386**	**40.9**	**1**	**DnaE1**	All except *Firmicutes* [*Bacilli*, *Erysipelotrychi* (1)], *Tenericutes*, *Thermotogae*, *Fusobacteria*, *Gemmatimonadetes*, *Ignavibacteria*
**282**	**29.8**	**2**	**DnaE1 + DnaE2**	*Acidobacteria*, *Actinobacteria*, *Bacteroidetes*, *Chloroflexi*, *Planctomycetes*, *Proteobacteria*, *Spirochaetes*, *Synergistetes*, *Thermodesulfobacteria*, *Verrumicrobia*, *Gemmatimonadetes* (1), *Ignavibacteria* (1), *Nitrospirae* (1), *Thermi* (1), *Firmicutes* [*Clostridia* (1)]
**40**	**4.2**	**3**	**DnaE1 + 2xDnaE2**	*Actinobacteria*, *Bacteroidetes*, *Proteobacteria*, *Spirochaetes*, *Acidobacteria* (1)
8	0.9	4	DnaE1 + 3xDnaE2	*Bacteroidetes*, *Proteobacteria*
3	0.3	2	2xDnaE1	*Actinobacteria*, *Deferribacteres* (1)
3	0.3	3	2xDnaE1 + DnaE2	*Actinobacteria*, *Proteobacteria* (1)
3	0.3	3	DnaE1 + DnaE2 + DnaEX	*Actinobacteria*, *Proteobacteria* (1)
2	0.2	2	DnaE1 + DnaEX	*Proteobacteria*
**66**	**7.0**	**2**	**PolC + DnaE1**	*Firmicutes* (*Clostridia*, *Negativicutes*), *Fusobacteria*
3	0.3	3	PolC + DnaE1 + DnaE2	*Firmicutes* (*Clostridia*)
6	0.6	3	PolC + DnaE1 + DnaEX	*Firmicutes* (*Clostridia*, *Negativicutes*)
1	0.1	3	PolC + 2xDnaE1	*Firmicutes* [*Clostridia* (1)]
1	0.1	4	PolC + DnaE1 + 2xDnaEX	*Firmicutes* [*Negativicutes* (1)]
1	0.1	4	2xPolC + DnaE1 + DnaEX	*Firmicutes* [*Clostridia* (1)]
**130**	**13.8**	**2**	**PolC + DnaE3**	*Firmicutes* [*Bacilli*, *Erysipelotrychi* (1)^a^], *Tenericutes*, *Thermotogae*
3	0.3	3	PolC + DnaE3 + DnaE2	*Firmicutes* [*Bacilli*, *Clostridia*(1)^b^]
5	0.5	3	PolC + DnaE3 + DnaEX	*Firmicutes* (*Bacilli*)
2	0.2	3	2xPolC + DnaE3	*Tenericutes* (1), *Thermotogae*(1)

Polymerase combinations observed in >1% of the analyzed bacterial genomes are emphasized by bold font. Relevant bacterial phyla (and classes of *Firmicutes* in parentheses) are listed in the last column [single occurrences are marked with ‘(1)’].

^a^*Erysipelotrychi* bacteria have low rRNA similarity to other *Firmicutes*, some phenotypic traits differ considerably. Previously these bacteria were classified with bacteria of current phylum *Tenericutes* (64).

^b^*Sulfobacillus acidophilus* is a member of sulfobacilli, which were only tentatively assigned as a family of *Clostridia* (64). According to genomic distribution and domain architectures of PolIIIαs, *S. acidophilus* is related to *Kyrpidia tusciae* and *Alicyclobacillus acidocaldarius*, both belonging to the *Bacilli* class. Previously, these three genera were classified together (64).

### Domain architectures

To better understand differences between distinct polymerase groups, we compiled their domain architectures (the composition and the arrangement of structural-functional domains). To this end, for every polymerase sequence we asked which of the conserved domains revealed by DnaE and PolC structures (Figure 1) as well as any other domains are present and in which order. The domain-mapping results are fairly robust as only in a relatively small number of cases we were unable to map any conserved domains with statistically significant values for polypeptide chain regions exceeding 50 residues. Moreover, regions without the domain assignment occur only at sequence termini.

**Figure 3.**
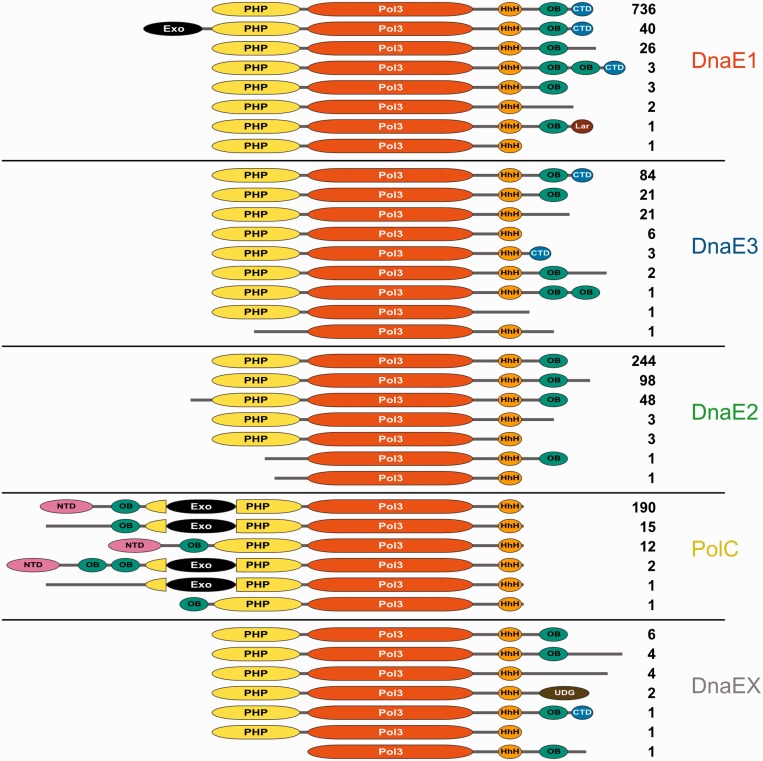
Domain architectures of distinct groups of C-family polymerases. Labels of standard polymerase domains are the same as in Figure 1. Nontypical domains: UDG, uracil DNA glycosylase; Lar, a domain related to the restriction alleviation protein Lar. Numbers indicate the total number of sequences having this particular domain architecture in the nonredundant set (1590 sequences).

The survey (Figure 3 and Supplementary Table S2) revealed that each polymerase group has a typical architecture. At the same time, we observed a significant variability within each group. Most often, new variants are associated with the loss of one or more domains, but there are also cases of the domain gain. The most conserved subset of structural-functional domains includes the combination of PHP, Pol3 and (HhH)_2_ domains. For the sequence to be annotated as a putative C-family DNA polymerase, we required that it should have the intact region harboring the polymerase active site. Therefore, the conservation of the polymerase core (Pol3) is expected. Somewhat surprisingly, PHP and (HhH)_2_ domains are also nearly universally present, suggesting their important structural and/or functional role.

The most common architecture of PolC (86%) is identical to that of *B. subtilis* PolC. It consists of NTD, OB domain, PHP with the inserted exonuclease (Exo) domain, Pol3 and the (HhH)_2_ motif (Figure 3). For a small number of PolC sequences (7%), we failed to assign NTD with statistically significant values. However, it has been observed that PolC NTDs are poorly conserved at the sequence level (18). Thus, it may well be that in most such cases a strongly diverged NTD is present in the unassigned N-terminal region. One of the hallmarks of PolC is the proofreading exonuclease (Exo) inserted into the PHP domain. Thus, it was surprising to find PolC sequences (6%) that lack the Exo insertion. Interestingly, all such PolC variants (with the single exception of an additional PolC copy in *Thermotogae*) were found exclusively in *Clostridia* and *Negativicutes*, two classes of *Firmicutes* bacteria. *Firmicutes* belonging to these two classes all have PolC paired with DnaE1 and not with DnaE3 (Table 1). An example of domain expansion, represented by a tandem duplication of OB domains, is observed in two PolCs from *Lactococcus* genus.

The most typical architecture of the DnaE1 group (91%) is represented by the *E. coli* PolIIIα subunit and includes PHP, Pol3, (HhH)_2_, OB and CTD domains. The PolIII proofreading exonuclease activity in *E. coli* is supplied by the separate ε-subunit complexed with α-subunit (1). Surprisingly, we detected a fraction of DnaE1 subunits, all of them in *Bacteroidetes*, having the exonuclease domain as part of the same polypeptide chain. Previously, this has been observed only in PolC type α-subunits. However, unlike in PolC, the DnaE1 exonuclease domain is not inserted into the PHP domain but attached to its N-terminus through a linker (∼60 residues). This difference indicates that the incorporation of the exonuclease domain into PolC and DnaE1 are unrelated evolutionary events. Other variants display differences in the region, C-terminal to the (HhH)_2_ motif. In some of these cases, the CTD could not be identified with statistically significant values within the unassigned C-terminal region. Since CTD, similarly to NTD of PolC, is poorly conserved, at least a number of CTDs may have escaped identification. One of the minor variants features domain expansion (duplicated OB domain) in the otherwise canonical architecture. Another variant is truncated right after the OB domain, thus excluding any possibility of the CTD presence. Also, a second DnaE1 copy from δ-proteobacterium *Desulfococcus oleovorans* (a typical DnaE1 is also present) has CTD replaced with a small domain related to the restriction alleviation protein Lar (PFAM family: PF14354), a predicted rubredoxin-like zinc binding domain.

The dominating architecture in the DnaE3 group is the same as in the case of DnaE1, but less typical. Only 60% of DnaE3s, compared with 91% of DnaE1s, have this architecture, and a larger fraction of DnaE3s lacks one or more domains at the C-terminus. As many as 16% are truncated after the OB domain (do not have CTD), and additional 4% lack both OB and CTD domains. There is even a variant with the excised OB domain but with CTD present. DnaE3s in *Tenericutes* all lack CTD, and only 40% of them have an identifiable OB domain. *Thermotogae* sequences, which show the strongest divergence within the DnaE3 group, all lack recognizable OB and CTD domains.

At the level of domain architecture, DnaE2 polymerases differ from both DnaE1 and DnaE3 in that not a single DnaE2 has the CTD. Although some have protruding C-terminal tails, disorder predictions suggest that they are mostly unstructured. Several DnaE2 sequences lack not only CTD but also the OB domain.

Of those DnaEs that were not assigned to one of the above groups (DnaEX), most have architectures already found in DnaE1-3 groups. In an unusual variant, found as an additional polymerase in some strains of *Yersinia pestis* and *Salmonella enterica*, OB and CTD domains are replaced with the archaeal-type uracil DNA glycosylase domain (65), a member of the PFAM family PF03167.

**Figure 4.**
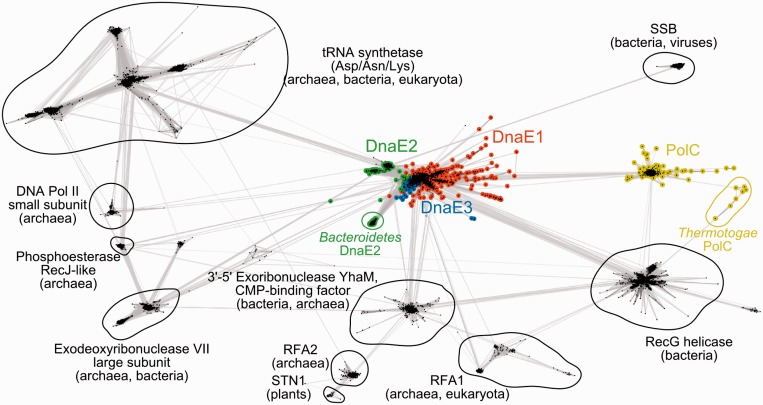
Homologs of PolIIIα OB domains clustered according to sequence similarity using CLANS (66). Each dot represents a single sequence. Stronger color intensity and shorter connecting lines correspond to the higher sequence similarity (according to *P*-value). Only the connections with *P*-value of 1e–09 or better are shown. Abbreviations: SSB, single-stranded DNA binding protein; RFA, replication factor A; STN1, a subunit of the single-stranded DNA binding CST complex, involved in telomere maintenance.

In addition to the PHP-Pol3-(HhH)_2_ conserved core, the OB domain is also nearly universally present (detected in at least 97% of polymerases). However, its position in relation to the conserved core is completely different: in DnaE, it is C-terminal, while in PolC, it is N-terminal. This observation inevitably raises a question: do OB domains of both PolC and DnaE derive from the ancestral C-family polymerase or had they been acquired independently by DnaE and PolC lineages? If the first scenario were true, DnaE and PolC OB domains would be expected to be closest to each other. Conversely, the second scenario would imply that DnaE and PolC OB domains should be more similar to their respective parental OB domain families than to each other. To distinguish between the two alternatives, we collected a large number of diverse homologs of both DnaE and PolC OB domains (up to 10 PSI-BLAST iterations, 1e–03 E-value cutoff) and clustered them according to all-against-all sequence similarities. Clustering revealed that DnaE OB domains are connected to a considerable number of OB domain families, while PolC OB domains are almost exclusively linked to OB domains of DnaE (Figure 4). These results imply that OB domain of PolC descended from the ancestral DnaE OB domain and not from any other source. In other words, the results suggest that the last common ancestor of DnaE and PolC already had the OB domain and was of the DnaE type, that is, with OB domain C-terminal to the polymerase core.

### Structure and surface conservation

**Figure 5.**
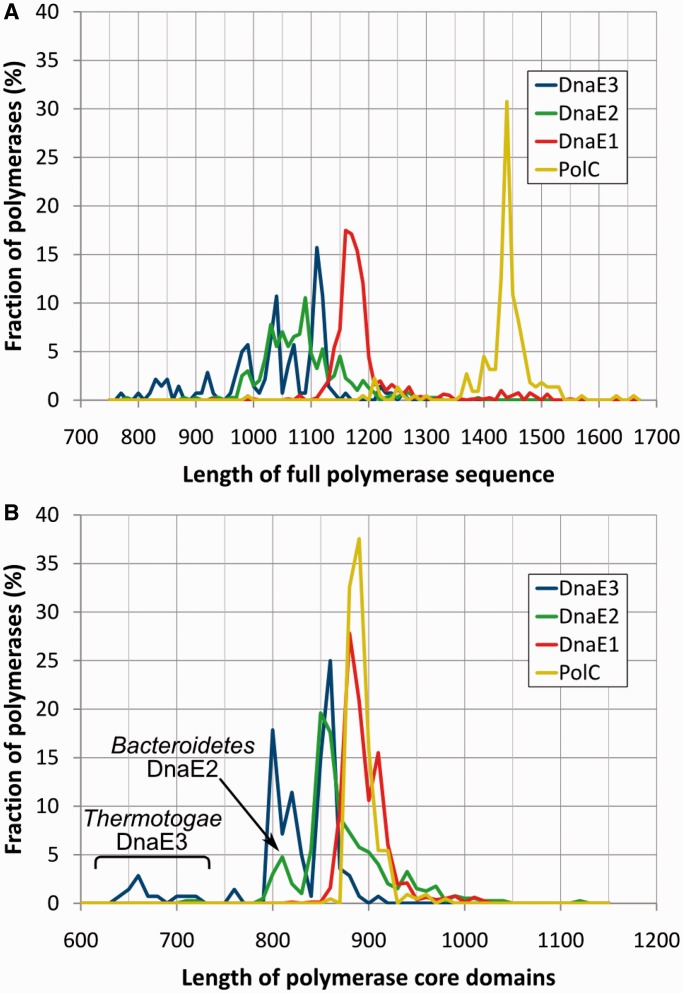
Polymerase sequence length distributions in four distinct groups. Distributions for (**A**) full-length sequences and (**B**) only the core region [PHP, Pol3 and (HhH)_2_ domains; residues 6–889 of *E. coli* DnaE1]. The vertical axis indicates the fraction of polymerases in a given length interval (at the step of 10 residues) for each group separately. DnaE3 of *Thermotogae* and DnaE2 of *Bacteroidetes* have distinctly shorter core sequence regions than other polymerases in corresponding groups.

The four polymerase groups differ considerably according to the sequence length (Figure 5A). Not surprisingly, PolC polymerases are the longest owing to the inserted exonuclease domain and a fairly long PolC-specific NTD. DnaE1 polymerases are typically longer than DnaE2 or DnaE3. Despite the large difference in the overall length, a common feature of PolC and DnaE1 polymerases is that their length varies in a fairly narrow range. In contrast, the length of both DnaE2 and DnaE3 polymerases shows strong heterogeneity. Our survey of domain architectures (Figure 3) suggested that the length variability to a large degree is determined by the absence/presence of structural domains in terminal regions. Therefore, we repeated the analysis of sequence length distribution for only the evolutionary conserved core consisting of PHP, Pol3 and (HhH)_2_ regions (Figure 5B). Strikingly, the core regions of PolC and DnaE1 have nearly identical length distributions. On the other hand, similarly to full sequences, the core regions of DnaE3 and DnaE2 are both shorter and considerably more heterogeneous. The heterogeneity of DnaE3s is mainly due to structural differences in PHP and ‘thumb’ domains. If taxonomy is considered, by far, the most distinct DnaE3 polymerases are present in *Thermotogae*. Their PHP domain is significantly smaller, as it lacks several structural elements (Supplementary Figure S1A). Similar, but less severe reduction of the PHP domain can be seen in DnaE3s of most *Tenericutes* and at least some *Firmicutes*. In addition to the degraded PHP domain, *Thermotogae* DnaE3 polymerases also feature a strongly reduced ‘thumb’ (Supplementary Figure S1B). Their ‘thumb’ lacks a helix-loop-helix motif, corresponding to *T. aquaticus* DnaE1 residues 513–552, that provides additional contacts with the DNA duplex (11) and may reach the downstream template DNA. Intriguingly, a similarly reduced ‘thumb’ that has been observed in the crystal structure of *G. kaustophilus* PolC (16) appears to be typical for PolC polymerases. The DnaE2 core, similarly to DnaE3, on average is shorter than that of PolC or DnaE1, but the reduced PHP and ‘thumb’ domains are mainly confined to DnaE2s of *Bacteroidetes*.

**Figure 6.**
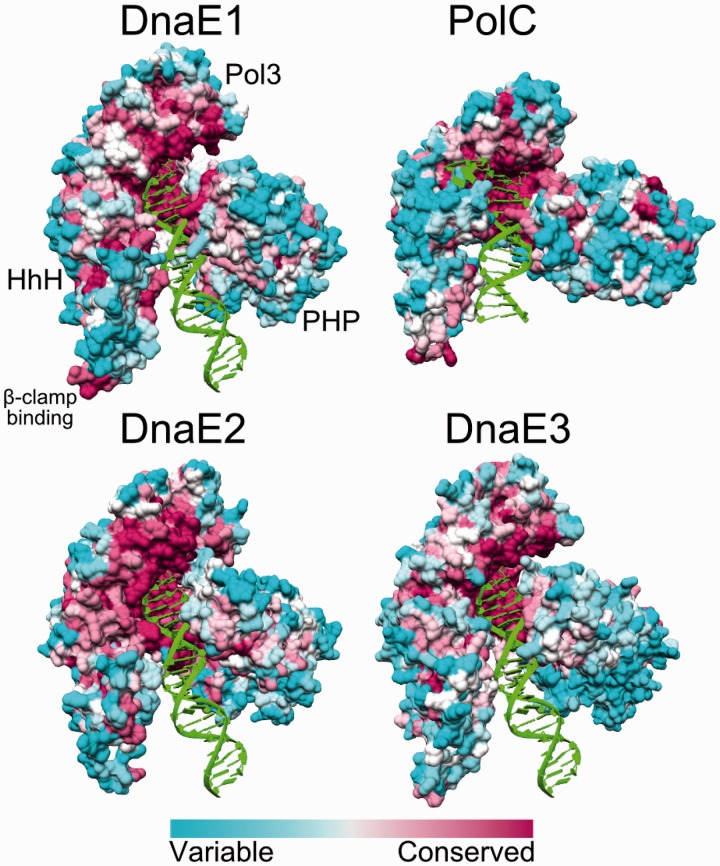
Surface residue conservation in different polymerase groups. The structures shown are *T. aquaticus* DnaE1 (PDB ID: 3E0D), *G. kaustophilus* PolC (PDB ID: 3F2B), *M. tuberculosis* DnaE2, *B. subtilis* DnaE3. All structures are shown in same orientation. OB domain and CTD were removed for clarity. Relative positions of all domains are indicated for DnaE1. The surfaces are colored according to ConSurf results: variable—cyan, conserved—maroon.

Protein surface is often as informative as the structure. Differences in patterns of surface conservation may indicate the relative importance of functional sites. It can be seen in Figure 6 that the active site and some of the DNA binding surfaces are highly conserved in all four polymerase groups. Strikingly, DnaE3 and even nonessential DnaE2 polymerases show the conservation as strong as the DnaE1 and PolC groups, representing main replicative polymerases. However, the β-clamp binding site is more strongly conserved in DnaE1 and PolC groups.

**Figure 7.**
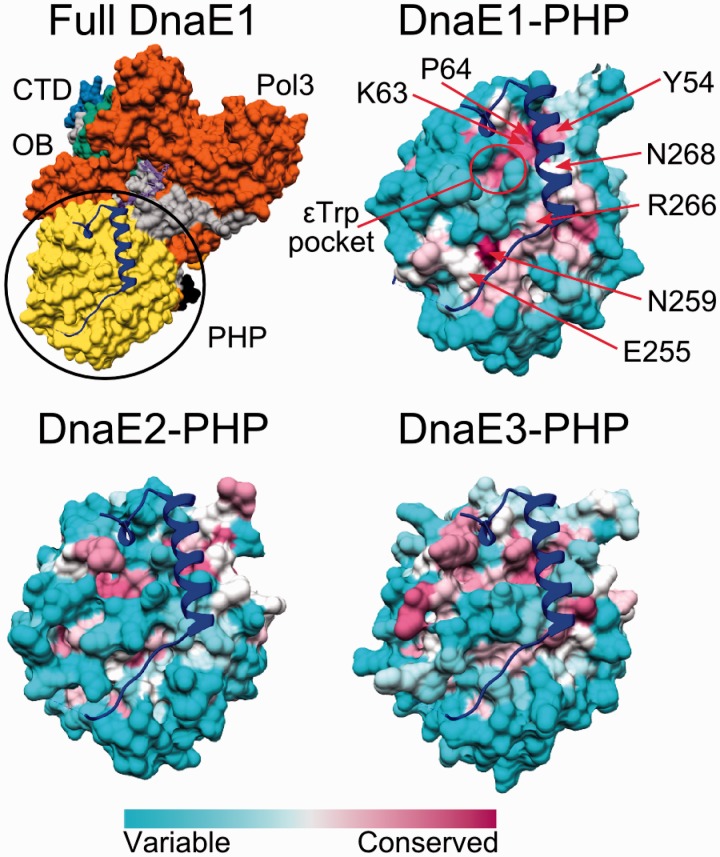
Surface conservation of the PHP domain at the putative ε-binding site in the three DnaE groups. Top left panel shows the full structure of *T. aquaticus* DnaE1, colored according to the domain organization. The position of the CTD of ε-subunit (blue ribbon) corresponds to that obtained for the *E. coli* DNA polymerase IIIα-ε chimera (PDB ID: 4GX9). Remaining panels show surface conservation of only the PHP domain for each of the three DnaE groups. PHP domains with the overlaid C-terminal segment of ε-subunit (blue ribbon) are shown in the same orientation as in the top left panel. Most conserved residues contacting the ε-subunit are indicated with red arrows. The pocket where Trp241 of the ε-subunit is bound is also indicated.

It has been known for some time that the PHP domain of *E. coli* PolIIIα (DnaE1) harbors the binding site of the proofreading ε-subunit (67). Recently, Ozawa *et al.* mapped this binding site by solving the crystal structure of the C-terminal part of ε-subunit fused to the PHP domain through a flexible linker (68). Our analysis of the PHP surface conservation shows that DnaE1 polymerases have a conserved patch in the exact position of the binding site of the ε-subunit (Figure 7). Conserved residues forming putative contacts with the ε-subunit can be easily identified along the whole length of contacting surface patch (Figure 7). Residues, maintaining the α-ε interaction in *E. coli* have been outlined according to the chimeric structure (68). At least two residues of the *E. coli* ε-subunit C-terminal segment (His225 and Trp241) were experimentally shown to be important for maintaining the interaction with α-subunit (69,70). εHis225 forms a hydrogen bond to Lys63 of the α-subunit, while εTrp241 is embedded in a conserved hydrophobic pocket. Both Lys63 (with the adjacent proline) and the εTrp-binding pocket are highly conserved in the DnaE1 group, but only moderately in DnaE3 polymerases. The rest of the putative ε-subunit binding surface patch in DnaE3 is even less conserved. Also, in structurally distinct *Thermotogae* DnaE3 polymerases, the missing structural elements in the PHP domain constitute a large part of the putative ε-subunit binding surface (Supplementary Figure S1C). The corresponding surface region in DnaE2 polymerases is not conserved at all, suggesting that they do not bind ε-subunit or at least not in the same way as *E. coli* DnaE1.

### Electrostatic properties

**Figure 8.**
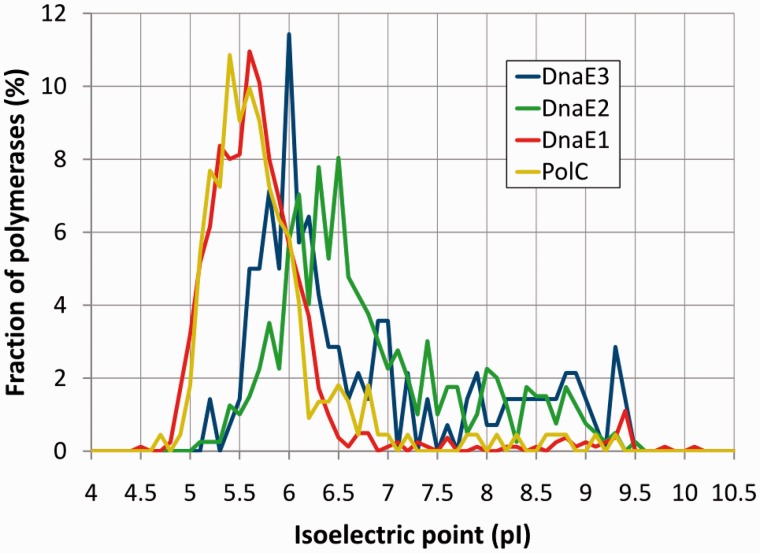
Predicted polymerase isoelectric point (pI) value distributions in four distinct groups. pI values were calculated for the core region [PHP, Pol3 and (HhH)_2_ domains; residues 6–889 of *E. coli* DnaE1]. The vertical axis indicates the fraction of polymerases at a given pI value (with a step of 0.1) for each group separately.

Owing to different functional roles, different polymerase groups might be expected to have distinct electrostatic properties. To make a proper comparison between polymerase groups, similarly to the length analysis, we only used the common conserved core [PHP, Pol3 and (HhH)_2_]. As a simple initial test, we computed theoretical isoelectric points (pI) for each protein. Despite simplicity of the approach, the pI calculation results revealed striking parallels with the results of sequence length analysis. Both the average pI values and the pI distribution for DnaE1 and PolC are almost identical (Figure 8). DnaE2 and DnaE3 groups both have on average higher pI values than DnaE1/PolC. Furthermore, in contrast to DnaE1/PolC, pI values for DnaE2 and DnaE3 display broad distribution. A more detailed analysis in different subgroups (Supplementary Figure S2) revealed that extreme pI values tend to coincide with major structural deviations. For example, DnaE3 polymerases of *Thermotogae* and *Tenericutes* featuring significant loss of structural elements have the highest pI values (averages of 7.9 and 7.6, respectively). In Figure 8, it can be seen that high pI values are also observed for a few DnaE1 and PolC polymerases. Interestingly, most of these ‘unusual’ DnaE1s and PolCs are found in bacteria with tiny (<1 Mb) AT-rich genomes (Supplementary Figure S3). DnaE1 polymerases with high pI values are almost exclusively found in insect symbionts, while PolCs are mostly found in mycoplasmas and phytoplasmas. In contrast, high pI values in DnaE3 and DnaE2 groups are not specifically associated with small genome size or low GC content.

The charge distribution on the surface of polymerases is unbalanced (Supplementary Figure S4). Typical DnaE1 and PolC polymerases with low pI (pI < 6) have positive charge patches predominantly in the DNA binding groove. The increase in pI values appears to be associated both with the increase in positive charge within the DNA binding groove and the nonspecific dispersal of positive charges. Comparison of several DnaE1/DnaE2 and PolC/DnaE3 pairs originating from the same species illustrates a more strongly pronounced positive charge of the DNA binding groove in DnaE2 and DnaE3 compared with the corresponding DnaE1 and PolC polymerases (Supplementary Figure S4). These observations suggest that typical DnaE1 and PolC polymerases bind the DNA less strongly than DnaE2 or DnaE3.

### Functional motifs

Sequence motifs may define important functional characteristics. Therefore, we analyzed how known functional motifs differ in distinct polymerase groups and asked whether there are some novel motifs.

#### Polymerase active site and its neighborhood

The polymerase active site aspartic residues [*E. coli* D401, D403 and D555 (7,71)] that coordinate catalytic magnesium ions are absolutely conserved in the entire C-family (Supplementary Figure S5). If we consider the immediate vicinity, PolC clearly differs from DnaE groups, which all display a similar conservation pattern. For example, additional absolutely conserved Asp (*E. coli*, D405) is present in all three DnaE groups, but is replaced by Asn (*B. subtilis*, N970) in PolC. DnaE2 appears to have slightly distinct active site neighborhood in comparison with either DnaE1 or DnaE3. However, the polymerase active site is highly conserved in all groups and it is unclear to what degree differences in the neighborhood are significant.

#### PHP metal binding site

**Figure 9.**
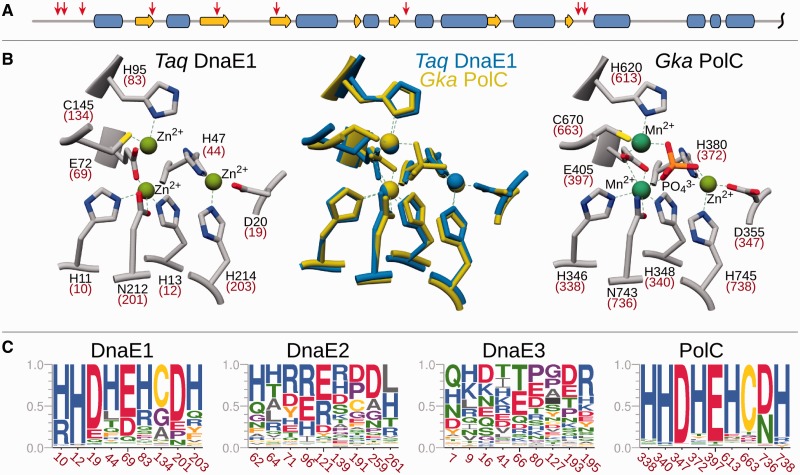
Conservation of metal binding residues in the PHP domain. (**A**) Schematic representation of the secondary structure for the PHP region (1–290) of *T. aquaticus* DnaE1. The positions of nine metal-coordinating residues are indicated with red arrows. (**B**) The structures of the PHP metal binding site in *T. aquaticus* DnaE1 (PDB ID: 4IQJ, left) and *G. kaustophilus* PolC (PDB ID: 3F2D, right) with residue names and numbers labeled. Corresponding residues in *E. coli* DnaE1 and *B. subtilis* PolC are indicated in parenthesis. The same superimposed structures are shown in the middle. (**C**) Sequence logo representation of the conservation of corresponding positions in different polymerase groups. Positions are labeled according to a representative from each group: *E. coli* DnaE1, *M. tuberculosis* DnaE2, *B. subtilis* DnaE3 and PolC.

In contrast to the highly conserved polymerase active site region, the PHP domain turned out to be much more informative. It has been associated with a novel Zn^2+^-dependent proofreading exonuclease activity in at least some C-family polymerases (11,72). We analyzed the conservation of nine PHP positions (Figure 9A and B) that in *G. kaustophilus* PolC and *T. aquaticus* DnaE1 crystal structures are involved in metal binding (11,16,73). Results show (Figure 9C) that these positions display distinct levels of conservation in different groups. PolC has all nine positions strongly conserved. Similar, albeit less strong, conservation is observed in the DnaE1 group. In contrast, the corresponding positions in DnaE2 and even more so in DnaE3, display little if any conservation.

Since polymerase groups, in particular DnaE1, span a variety of bacterial lineages, we also looked at the PHP metal binding site in more detail, taking into account bacterial taxonomy (Supplementary Figure S6). It turned out that DnaE1 sequences of three major classes of *Proteobacteria* (α, β and γ) all have extensive substitutions in the PHP active site. Notably, not a single DnaE1 in these bacteria possesses all nine (or even eight) residues from the consensus pattern. This finding is consistent with previous studies suggesting that at least some of *Proteobacteria* might have an inactivated PHP exonuclease active site (74,75). DnaE1 polymerases in *Bacteroidetes* and *Fusobacteria* also display a significant variability in the PHP metal binding positions. In contrast, DnaE1 polymerases in *Firmicutes* and all of the remaining bacteria, including *δ-* and *ε-proteobacteria*, display extremely high conservation of the PHP metal binding site. Although as a group, DnaE2 polymerases do not have the conserved canonical pattern, a small fraction of DnaE2s have the PHP active site mostly intact. These polymerases belonging to a subgroup of *Actinobacteria* (including *M. tuberculosis*) appear to form a distinct clade in the phylogenetic analysis. On the other hand, none of the DnaE3 subgroups showed any conservation, suggesting that the defective PHP metal binding site is a hallmark of DnaE3 polymerases.

Potentially, DnaE polymerases could have two different types of exonuclease activity, one due to the bound ε-subunit and the second due to the exonuclease activity of the PHP domain. An interesting question is whether the two exonuclease activities are mutually exclusive? Recently, it was suggested that they likely might be (75). In such case, it might be expected that DnaE1 polymerases with the nonfunctional PHP metal binding site would maintain a conserved ε-binding site, while those with the functional PHP active site would not. We find that *Proteobacteria* DnaE1 polymerases, featuring extensive substitutions in the PHP active site (Supplementary Figure S6), indeed have a strongly conserved putative ε-binding site (Supplementary Figure S7). Nonetheless, DnaE1s of other bacteria with the perfectly conserved PHP active site (Supplementary Figure S6) still retain considerable surface conservation (Supplementary Figure S7). In other words, the presence of the intact PHP metal binding site does not seem to preclude the ε-subunit binding. Apparently, the differences between DnaE groups are more relevant. For example, DnaE-type polymerases in *Firmicutes*, partitioned into DnaE1 and DnaE3 groups (Figure 2), show the opposite trends (Supplementary Figure S7). These DnaE1 polymerases have both the intact PHP metal binding site and a strongly conserved putative ε-binding surface patch. In contrast, DnaE3s have both the disrupted metal binding site and almost no conservation of the PHP surface.

#### DNA sliding clamp binding motif

**Figure 10.**
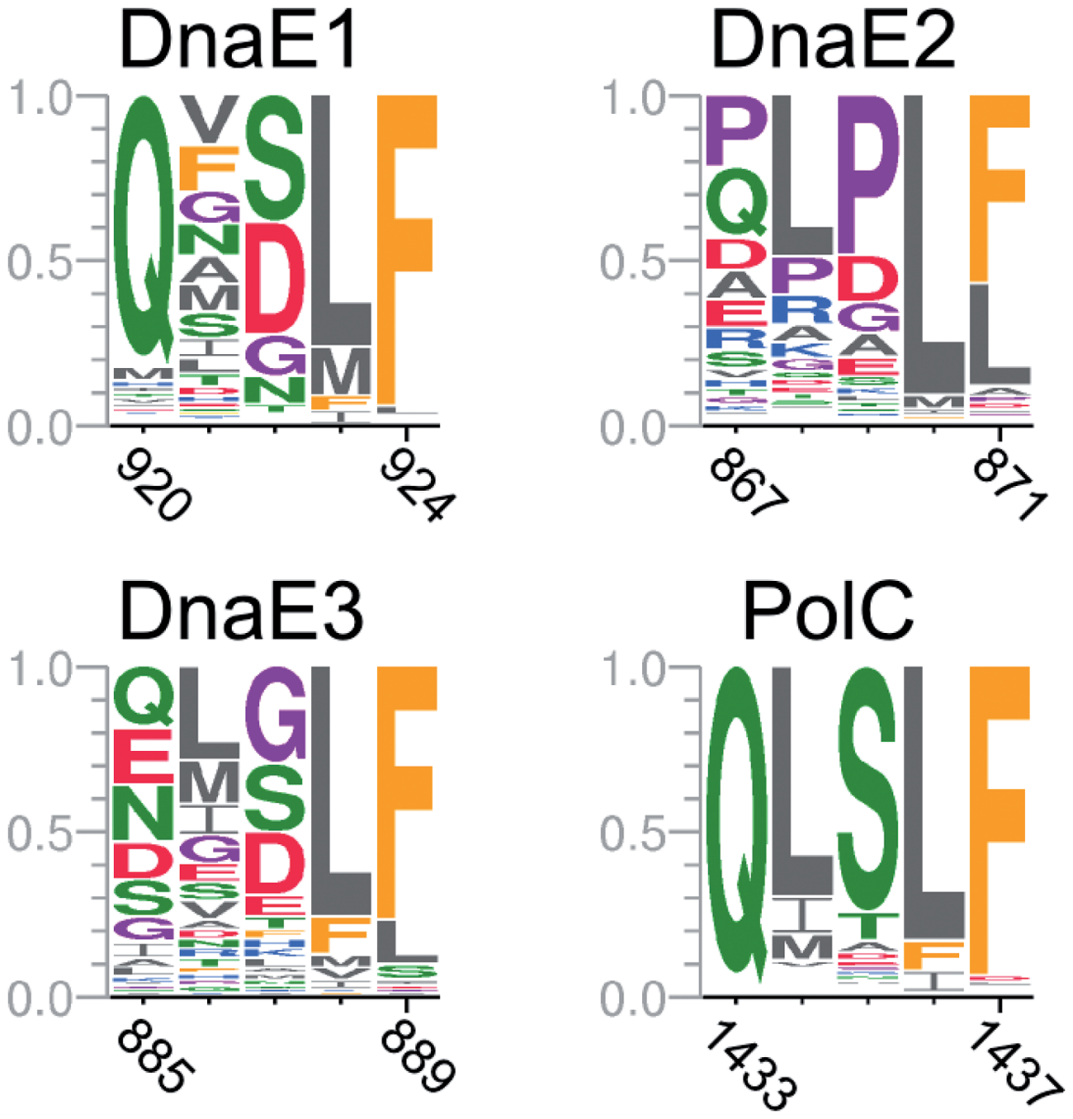
The β-clamp binding motif in different polymerase groups. Residues are labeled according to the same representatives as in Figure 9.

Bacterial replicative DNA polymerases interact with the DNA sliding clamp to achieve high speed and processivity. The interaction is mediated by a short sequence motif within PolIIIα subunit. The consensus β-clamp binding motif has been identified previously as the pentapeptide QL[S/D]LF (76) in which positions 1, 4 and 5 appear to be the most important (77,78). We found that the presence and nature of the β-clamp binding motif vary considerably between different polymerase groups (Figure 10). The PolC group almost universally (98% of sequences) features the consensus motif QLSLF that is closest to the previously established consensus β-clamp binding motif. The β-clamp binding motif, also present in nearly all (99%) DnaE1 sequences, is less conserved than in PolC, yet is still close to the consensus. In particular, the first (Q) and the last two positions (LF) are well conserved. In contrast, we identified a putative motif in only 75% of DnaE3 polymerases. Furthermore, only the positions 4 and 5 of the motif are conserved. DnaE2 sequences are also heterogeneous in respect to the presence of the β-clamp binding motif. About 85% of DnaE2s have an identifiable motif, but it is visibly different from the canonical one. More specifically, positions 1 and 3 are dominated by proline, which is not present in corresponding positions in other polymerase groups.

Like in the case of PHP metal binding site, a more detailed look at taxonomy-based β-clamp binding motifs revealed significant variation (Supplementary Figure S8). For example, DnaE1 polymerases in *Deferribacteres* (five sequences) and a small fraction of *Bacteroidetes* (five sequences) do not have a recognizable β-clamp binding motif at all. In these bacteria, DnaE1 appears to be the primary replicative polymerase, raising a question of how the replication processivity is achieved in those cases. Notably, DnaE1 sequences that co-occur with PolC in *Clostridia* (*Firmicutes*) have a motif typical to other DnaE1 sequences and not to the ‘weak’ motif of DnaE3 (also co-occurring with PolC) (Supplementary Figure S8). A small fraction of DnaE3 sequences (14%), most of them in bacteria of the order *Bacillales*, also have motifs similar to those of DnaE1 or PolC (QxxLF, where x is any residue). *B. subtilis* is one of these bacteria and its DnaE3 has a relatively ‘strong’ motif (QMGLF).

Several different putative β-clamp binding motifs can be identified in DnaE2 polymerases. The distinct motif (PLPLF) is predominantly found in *α-proteobacteria*. A similar consensus motif (xLPLF) is typical for *γ-proteobacteria*, but *β-proteobacteria*, although taxonomically close to *γ-proteobacteria*, seem to have a different and even ‘weaker’ consensus motif, xxxLL. Some *Actinobacteria* seem to have a nontypical QLPLx motif, which in almost half of the cases can be extended to the hexameric QLPLxL motif, similar to the consensus motif of Hda, DnaA-related protein (77,79).

#### Putative protein–protein interaction motif in DnaE2

In addition to the known functional sites, we also searched for any other conserved motifs that stand out in at least one of the groups. One such motif is associated with ∼77% of DnaE2 sequences. This motif, noticed previously (35), features the SRDF[H/R] consensus sequence at the very C-terminus (Supplementary Figure S9A). The motif is part of the region, predicted to be intrinsically unstructured (Supplementary Figure S9B). High conservation combined with the lack of a defined structure typically is the signature of a protein–protein interaction motif, but in this case its specific function remains to be established. This motif is found in the majority of DnaE2s of *Proteobacteria* (except most of *δ-proteobacteria*), part of *Actinobacteria* (including *M. tuberculosis*), a few *Bacteroidetes* and some minor phyla. Interestingly, all of these DnaE2 sequences clustered together in the phylogenetic tree (bootstrap value 97%), despite the omission of the motif during the tree construction.

### Polymerase combinations and global characteristics of the bacterial cell

Our analyses presented above indicate that there are significant differences between distinct groups of C-family polymerases. In addition, both the number and the type of polymerases encoded in genomes vary considerably. It might be expected that different polymerase combinations may represent different functional capabilities pertaining to DNA replication such as speed and mutation bias and/or dealing with oxidative stress exerted onto DNA. Therefore, we asked whether polymerase combinations correlate with global characteristics of bacterial species such as the genome size, genome GC content and the use of oxygen.

**Figure 11.**
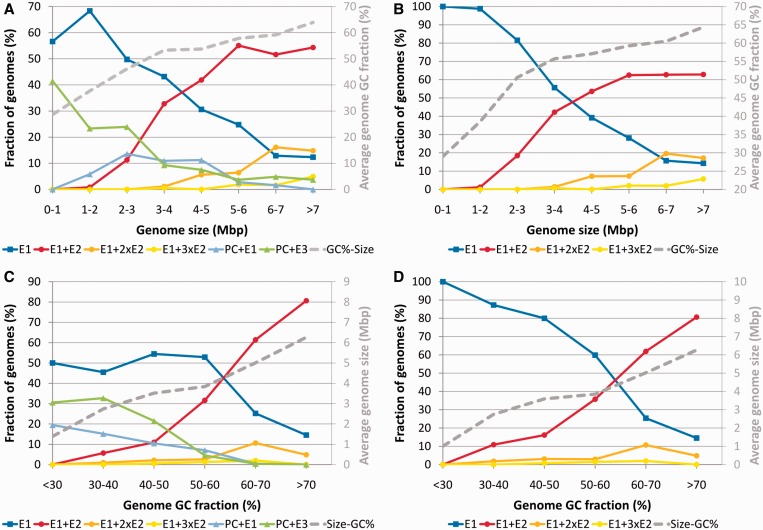
The relationships between different polymerase combinations and genome size (**A** and **B**) or genomic GC content (**C** and **D**). Data for all genomes (A and C) and genomes that do not encode PolC (B and D) are depicted separately. The remaining nontypical polymerase combinations are incorporated into those shown. The relationships between genome size and genomic GC content (where one component is binned and the other component is the calculated average for each bin) are depicted as secondary graphs (gray). ‘E’ indicates DnaE, ‘PC’, PolC.

First, we looked at the genome size. We divided genomes into several bins according to their size and asked which fraction of genomes in each bin has a particular polymerase combination. The results revealed several clear trends (Figure 11A and B). Except for the smallest genomes, the fraction of bacteria carrying sole DnaE1 decreases as genomes become larger. At the same time, the fraction of genomes that, in addition to DnaE1, encode one or more DnaE2 increases dramatically (Figure 11A). A steady decrease with the increase in genome size is also observed for the fraction of genomes represented by the PolC and DnaE3 combination. However, PolC-carrying bacteria are restricted to only a few phyla. Therefore, we also looked at only those genomes that do not have PolC and are distributed throughout the bacteria kingdom. The picture does not change significantly, yet the opposite trends corresponding to DnaE1 alone and DnaE1 accompanied by one or more DnaE2 become clearer (Figure 11B).

Next, we looked at the GC content of bacterial genomes. It varies from 14% for *Candidatus Zinderia insecticola*, a member of *β-proteobacteria* (80) to 75% for actinobacterium *Cellulomonas fimi* (81). As in the case of genome size, we grouped genomes into several bins according to the GC content and looked at the spectrum of polymerase sets represented in each bin. Again, similarly to the genome size analysis, there is a clear trend (Figure 11C). The fraction of genomes with single DnaE1 is about the same up to ∼60% GC. For GC-rich genomes (>60% GC) it decreases, while the fraction of those coding for DnaE2 in addition to DnaE1 increases dramatically. Combinations that include PolC and either DnaE3 or DnaE1 essentially disappear from genomes with the GC content >60%. If we consider only non-PolC genomes (Figure 11D), the picture becomes similar to that of dependency on the genome size (Figure 11B). The only difference is that the increasing number of DnaE2 polymerases is associated with the increasingly larger genome size, but the increase of GC seems to coincide only with the presence and not the number of DnaE2s.

**Figure 12.**
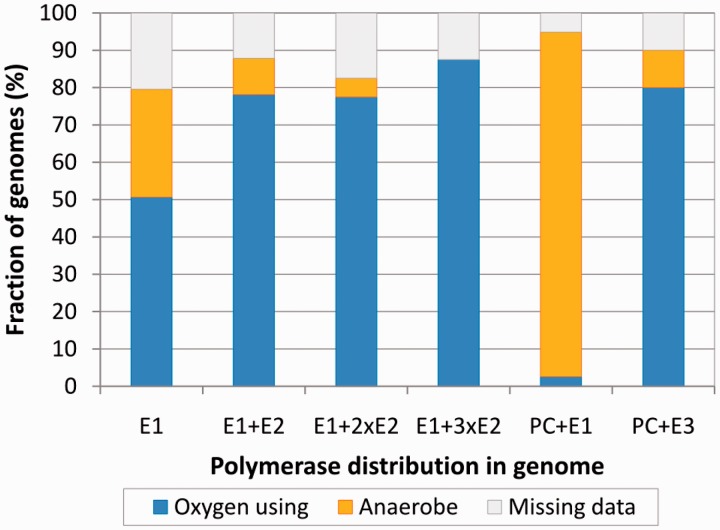
The relationship between different polymerase combinations and the use of oxygen by corresponding bacteria. Bacterial species (genomes) are divided into three categories: (i) oxygen using, including aerobes, facultative aerobes and microaerophiles (blue), (ii) anaerobes (yellow) and (iii) those for which data regarding the use of oxygen is unavailable (gray).

The use of oxygen in bacterial metabolism is associated with the oxidative damage to the DNA. We therefore asked whether the aerobiosis has any correlation with the polymerase combinations in genomes. We divided bacteria into two broad groups. The first group, oxygen-using bacteria, included aerobes, facultative aerobes/anaerobes (preferentially use oxygen if available) and microaerophiles (require oxygen, but only at low levels), while the second group consisted of anaerobes. Genomes with DnaE1 as a sole C-family polymerase are common for both groups (Figure 12). However, bacteria that have DnaE1 and any number of DnaE2s are almost exclusively oxygen using. Interestingly, there is a sharp contrast between the two groups of PolC-carrying bacteria. Those that have PolC + DnaE1 nearly all are anaerobes, while those having PolC + DnaE3 are predominantly oxygen-using bacteria.

Taken together, these results show that specific polymerase sets encoded in a given genome strongly correlate with genome size, GC content and oxygen requirement. In particular, it appears that the presence of DnaE2 together with DnaE1 is linked to bacteria featuring large GC-rich genomes and living in aerobic environments. However, this does not necessarily imply the causal relationship as genome size and GC content are also correlated to each other (secondary axes in Figure 11). We therefore sought any other data that would either support or contradict the idea that the presence of DnaE2 might influence genome properties (size, GC content or both). To this end, we analyzed whether there is any correlation between electrostatic (pI) and structural (length) properties of DnaE2 polymerases and either genome size or GC content (Supplementary Figures S3 and S10). The strongest correlation (Spearman’s rank correlation coefficient ρ = 0.58) was observed between the length of the DnaE2 polymerase core (PHP, Pol3 and (HhH)_2_) and GC content of the genome, while no correlation was found with the genome size (Supplementary Figure S10). Moreover, no significant correlation with either GC content or genome size was observed for DnaE1, DnaE3 or PolC polymerases. The variation in sequence length of DnaE2 polymerases is mainly due to additional structural elements or deletions in the PHP and ‘thumb’ domains. Functional importance of these differences is not obvious, but the correlation with GC content provides another hint for the possible involvement of DnaE2 in shaping genomic GC content.

## DISCUSSION

**Figure 13.**
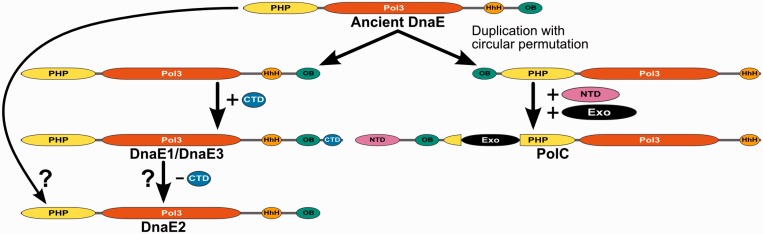
Proposed evolutionary pathway leading from the last common ancestor to extant groups of C-family DNA polymerases.

Our results based on a representative set derived from almost two thousand genomes showed that all bacteria have at least one C-family DNA polymerase. This strongly suggests that members of this family are principal genome replication enzymes throughout the bacterial world. C-family polymerases come in two major forms, DnaE and PolC, inferred to have evolved by ancient duplication (17). Since DnaE and PolC differ in the exact domain composition and arrangement, an interesting question is the nature of the ancestral form and the pathway that led to two extant forms. Our results combined with the available DnaE (7,8) and PolC (16) crystal structures indicate that both forms have a common universally conserved region that includes PHP, polymerase core (Pol3) and (HhH)_2_ domains. One other domain present in almost all members of C-family is the OB domain, which, however, in DnaE and PolC is attached to the opposite ends of the universally conserved region. Our results suggest that the OB domain of PolC derives from the DnaE-type OB domain (Figure 4). The simplest scenario, consistent with the common descent and the opposite location of OB domains in DnaE and PolC, involves the duplication with circular permutation of an ancestral DnaE-type polymerase (Figure 13). This scenario further implies that additional terminal regions have been independently acquired in PolC and DnaE lineages after the duplication. Again this is consistent with the observation that terminal regions are unique in PolC and DnaE1/DnaE3 (18). The analysis of coevolution patterns of DnaE and PolC polymerases (82) supports the ancient DnaE hypothesis. It was shown that PolC coevolved with some genes of the RNA degradation pathway, found exclusively in PolC-containing bacteria, while DnaE coevolved with proteins found throughout all bacterial phyla (82). After the emergence, PolC has apparently evolved as a highly specialized DNA polymerase that has to be complemented with a DnaE-type polymerase to form a fully functional replicase. In contrast, DnaE has evolved into different groups having different functional capabilities. DnaE1 is the most versatile as it is the only type of C-family polymerases that can replicate genome by itself (as in *E. coli*). DnaE3 is never found alone in any genome (always with PolC), indicating that it is specialized to complement the PolC function. DnaE2 is also never found alone consistent with its role of a nonessential polymerase, not involved in bulk DNA replication (26–32).

DnaE2 provides a clearest link between the domain architecture and functional specialization. None of DnaE2 polymerases have the CTD found in both DnaE1 and DnaE3 groups. Using mutational studies, genetic screens (2,78) and X-ray crystallography (73), it has been shown that CTD is important for binding Pol III τ-subunit. Pol III τ-subunit is part of the clamp loader complex and also a central coordinator of the replisome, as it interacts with both the replicative helicase and the α-subunit of Pol III. Therefore, the absence of the τ-interacting domain (CTD) implies that DnaE2 polymerases do not interact with τ-subunit (at least not in the same way as DnaE1). Interestingly, the domain composition of DnaE2 coincides with that of the presumed ancestral form of the C-family polymerase (Figure 13). It may be that DnaE2 is a direct descendant; an alternative possibility is that it had evolved from DnaE1/DnaE3 by subsequent loss of the CTD.

DnaE1 and DnaE3 groups are difficult to resolve by phylogenetic analysis, and the typical domain organization is the same for both. Therefore, it is puzzling that the functional versatility of these two groups is significantly different: DnaE1 can function by itself, while DnaE3 has to be always accompanied by PolC. A closer comparison of domain architectures reveals a significant heterogeneity within the C-terminal part of DnaE3 compared with DnaE1 polymerases. A significant fraction of DnaE3s do not have CTD or even the OB domain. One of the possible explanations of the observed heterogeneity is that OB and CTD domains are not critically important for the DnaE3 function. At least for CTD there is experimental evidence that its role is different in *E. coli* DnaE1 and *B. subtilis* DnaE3. Both proteins have identical domain architectures. In *E. coli*, CTD is responsible for the formation of a stable complex between DnaE1 and the clamp loader τ-subunit (78,83). This interaction retains DnaE1 within the replisome. In contrast, *in vivo* experiments in *B. subtilis* showed that the retention of DnaE3 at the active replication fork is entirely dependent on the interaction with the C-terminal tail of the single-stranded DNA binding protein (SSB) (84). These data indicate that *B. subtilis* DnaE3 either does not bind τ-subunit at all, or that this interaction is too weak in the absence of SSB. Unlike DnaE3, the retention of *B. subtilis* PolC at the replication fork does not depend on SSB (84), supporting the proposed role of its NTD for interaction with the clamp loader (18).

Conservation of sequence or structure motifs provides additional hints regarding functional differences. In this regard, the PHP domain proved to harbor the most distinguishing features of the four polymerase groups. One of the known functions of the PHP domain is the binding of the ε-subunit, which is the primary proofreader in the replisome of Gram-negative bacteria (1). Recently, the structure of a chimera composed of the PHP domain of *E. coli* DnaE1 and the ε-subunit has been determined, revealing the exact interaction site between these two subunits (68). This enabled us to ask how well the corresponding putative ε-subunit binding site is conserved among different polymerase groups. DnaE1 polymerases show high conservation all over the putative interaction site. On the other hand, DnaE3 and even more so DnaE2 polymerases show little conservation (Figure 7 and Supplementary Figure S7), implying that these polymerases most probably do not bind an ε-subunit, at least not in a similar way as *E. coli* DnaE1. However, separate (or integral, in the case of PolC) exonuclease might be not the only means to harbor exonuclease activity by C-family polymerases. At least in some polymerase α-subunits, the PHP domain has been found to be associated with a Zn^2+^-dependent proofreading activity (11,72). But are these just some unique cases or might the PHP-dependent proofreading activity be more widespread? PHP domains of PolC and DnaE1 (with some exceptions including three major classes of *Proteobacteria*) show highly conserved metal binding site (Figure 9 and Supplementary Figure S6). In contrast, DnaE3 polymerases show no conservation of corresponding positions whatsoever. Most of DnaE2 polymerases except a subgroup of *Actinobacteria* also lack the intact metal binding site. Therefore, our results suggest that DnaE3 and most of DnaE2 polymerases are devoid of proofreading activity. This is the actual case for *B. subtilis* DnaE3, for which no exonuclease activity could be detected (85). Perhaps the inferred lack of any exonuclease activity might also be linked to the inherently low fidelity observed for some DnaE3 polymerases (22,85).

Another difference between polymerase groups emerges if we consider the β-clamp binding motif. This motif mediates the interaction between a DNA polymerase and the DNA sliding clamp, thereby dramatically increasing processivity of the polymerase. The β-clamp binding motif in PolC polymerases is close to the ideal consensus, which is also one of the most potent β-clamp binding variants (76,78). The consensus motif in DnaE1 sequences shows higher variability, but is still reasonably conserved (except the second position). In contrast, in the DnaE3 group, only two last hydrophobic positions are conserved. Moreover, only about three quarters of DnaE3 sequences have this ‘weak’ β-clamp binding motif. Notably, some DnaE3 polymerases such as the one in *B. subtilis* do contain relatively ‘strong’ β-clamp binding motifs. However, considering the overall low conservation of β-clamp binding motifs in other DnaE3 polymerases, the *B. subtilis* case seems to be more of an exception rather than a rule. Although most DnaE2s do have the β-clamp binding motif, it is noncanonical with only last two positions showing a conservation pattern typical for other C-family polymerases. The observed low conservation of β-clamp binding motif in DnaE3 and DnaE2 groups suggests that there is little evolutionary pressure to retain a strong interaction with β-clamp.

Some interesting observations can be made from our survey of structural and electrostatic properties. Despite differences in domain architecture and functional versatility, PolC and DnaE1 share some remarkably similar features. Both polymerase groups have nearly identical length and narrow variability of the evolutionary core. Members of both groups also have a generally negative surface charge with positive charge being located mainly in the DNA binding groove. Similarly to the length of the core, the charge variance within these two replicative polymerase groups is rather low. Although the DnaE3 group also represents essential replicative polymerases, a large heterogeneity in both polymerase core length and surface charge distribution makes it more similar to nonessential DnaE2 polymerases. Moreover, the surfaces of the latter two groups are generally more positively charged than those of DnaE1 or PolC, suggesting a stronger DNA binding. Perhaps the elevated positive charge, in addition to the lack of exonuclease activity, might also contribute to the observed mutagenic character of DnaE2 and DnaE3 polymerases (22,26,34,85).

Genomic distribution of specific sets of polymerases revealed a picture, similar to that obtained with a smaller number of complete genomes (36). DnaE1, either as a single polymerase or in combination with one or more DnaE2s, is present in over three quarters of bacterial genomes that represent a variety of bacterial phyla. Another significant presence is made by PolC accompanied by either DnaE3 or DnaE1. However, this particular combination is typical for a narrow phyletic group, which mostly consists of low-GC Gram-positive bacteria. Taking this narrow distribution into account, it may not be so surprising that DnaE3s are absent from DnaE1-containing genomes. Perhaps more surprising is the observation that despite their wide dispersal, DnaE2s are infrequently found in PolC-containing genomes. DnaE2s are typically found as part of SOS-inducible mutagenic cassette identified in many bacterial genomes (27,34). At least some DnaE3s are also error-prone (22,85) and SOS-inducible (85). A possible explanation of the DnaE2 avoidance in PolC + DnaE3 genomes is that it might be disadvantageous for bacterium to have two related SOS-inducible polymerases.

Summing up all the observations about different polymerase groups, three distinct replication systems corresponding to DnaE1, PolC + DnaE3 and PolC + DnaE1 polymerase sets could be outlined (Table 2). The first two systems are represented by *E. coli* and *B. subtilis*, respectively. In *E. coli*, the DnaE1 polymerase performs chromosomal DNA synthesis all by itself (19). In *B. subtilis*, PolC does the bulk DNA synthesis of both DNA strands, but needs help from DnaE3 to extend RNA primers (24). The PolC + DnaE1 variant is intriguing, as it contains seemingly two highly efficient polymerases capable of bulk DNA synthesis. Unfortunately, there appears to be no experimentally characterized PolC + DnaE1 systems, despite their presence in prominent pathogens like *Clostridium difficile* or *Clostridium botulinum*. DnaE1 polymerases co-occurring with PolC have a typical β-clamp binding motif, a conserved putative ε-subunit binding surface and a perfect PHP metal binding site, all the properties of DnaE1 that can replicate the entire genome by itself. Could it be that normally DNA is replicated by DnaE1 and that PolC is involved only in specific situations? Such a view is at least partially supported by the observation that, although the majority of *Clostridia* (and *Negativicutes*) have both PolC and DnaE1 polymerases, some have only a single DnaE1. Furthermore, all PolC lacking the integral exonuclease are only found in clostridial genomes. Alternatively, it may be that PolC and DnaE1 work together at the replication fork and that the replication process is even more complex. Experimental approaches are clearly needed to gain knowledge on how the PolC + DnaE1 replication system really functions.

**Table 2.** Summary of three distinct replication systems

Polymerase set	**DnaE1**	**PolC + DnaE3**		**PolC + DnaE1**	
Representatives	*E. coli*, *T. aquaticus*, *M. tuberculosis*	*B. subtilis*, *Staphylococcus aureus*, *Streptococcus mutans*		*Clostridium difficile*, *Clostridium botulinum*, *Clostridium tetani*	
Polymerases	DnaE1	PolC	DnaE3	PolC	DnaE1
Domain architecture	Typical	Typical	Nonconserved retention of OB and CTD domains	Typical	Typical
PHP metal binding site	Conserved (except *α-,β-, γ-proteobacteria*, *Bacteroidetes*)	Conserved	Disrupted	Conserved	Conserved (except *Fusobacteria*)
β-clamp binding motif	Conserved	Conserved	Less conserved, heterogeneous	Conserved	Conserved
Putative proofreading exonuclease subunit (ε) binding site	Conserved (internal exonuclease domain in some *Bacteroidetes*)	Internal exonuclease domain	Mostly variable	Internal exonuclease domain (except in 16%)	Conserved
Electrostatic properties	Positive charge mainly in DNA binding cleft. Low variability.	Positive charge mainly in DNA binding cleft. Low variability.	Generally higher positive charge. High variability.	Positive charge mainly in DNA binding cleft. Low variability.	Positive charge mainly in DNA binding cleft. Low variability.
Role in the replisome	Bulk DNA synthesis of both strands (*E. coli*).	Bulk DNA synthesis of both strands, no extension of RNA primers (*B. subtilis*).	Only short extension of RNA primers (*B. subtilis*).	**Unknown**	**Unknown**

Based on our proposed evolutionary schema (Figure 13), it is tempting to speculate that the PolC + DnaE1 replication system might be most similar to an ancestral two-polymerase system. Consequently, *Firmicutes Clostridia* would be placed at the root of bacterial evolution. This is supported by the observation that two different replication systems are found in *Clostridia*: PolC + DnaE1 and a single DnaE1. Deep branching of *Firmicutes* [and in particular class *Clostridia* (86)] hypothesis is not novel and has been proposed earlier (86,87). The analysis of both replication initiation in Gram-positive bacteria and coevolution patterns of DnaE and PolC polymerases also suggest *Firmicutes* to be the most ancient bacteria (82,88). If this is the case, the evolution of C-family polymerase systems might have taken two different paths: toward the single DnaE1 (by losing PolC) and toward the PolC + DnaE3 system (DnaE1 evolving into DnaE3 due to the relaxed selection pressure on several of its functions).

There have been previous reports suggesting the involvement of PolIIIα subunits in shaping global properties of the genome such as the GC content (89,90). To further address this issue, we investigated the relationship between the polymerase combinations and well-defined global cell properties, namely genome size, GC content and the use of oxygen. For all of these properties, we identified clear trends. However, since PolC + DnaE3/DnaE1 combinations are found in a phylogenetically narrow group of bacteria, we mostly focused on DnaE1/DnaE1 + DnaE2 genomes that are not confined to specific bacterial phyla. Nonetheless, it is worth pointing out that PolC carrying bacteria are sharply divided according to the use of oxygen. PolC + DnaE1 combinations almost exclusively are anaerobes, whereas PolC + DnaE3 are typical for oxygen-using bacteria. Whether this division is based on functional differences of DnaE1 and DnaE3 or is just a consequence of a narrow phylogenetic distribution remains an open question. In DnaE1/DnaE1 + DnaE2 genomes, we found a clear dependence between the presence of DnaE2 and the increase of both the genome size and the GC content. Oxygen-using bacteria also tend to have DnaE2 more often. In other words, DnaE2 seems to be typical of oxygen-using bacteria having large GC-rich genomes. Genome size and the genomic GC content are the result of combination of various endogenous cell processes, environmental factors and selection pressure. Therefore, the relationship that we observed might simply be coincidental with some other important factors. In particular, it is well-known that there is a correlation between the genome size and the GC content so that large genomes are typically GC-rich (91–94). However, mutational spectra of replicative or repair DNA polymerases may directly contribute at least to the variation of the GC content. Although by now it is generally accepted that the overall mutation bias in bacteria is toward the lower GC content (95–97), there is a possibility that DnaE2 might contribute in offsetting or even reversing this bias. Supporting this idea is our observation that the length of DnaE2 polymerases is correlated with the GC content but not the genome size and that none of the groups of essential replicative polymerases shows similar correlation.

## SUPPLEMENTARY DATA

Supplementary Data are available at NAR Online.

## FUNDING

Research Council of Lithuania [MIP-079/2011]. Funding for open access charge: European Community’s Seventh Framework Programme [FP7-REGPOT-2009-1] project ‘MoBiLi’.

*Conflict of interest statement*. None declared.

## Supplementary Material

Supplementary Data
