# Supplementary material for: Comprehensive analysis of DNA polymerase III α subunits and their homologs in bacterial genomes
Source: Nucleic Acids Res. 2013 Oct 6;42(3):1393–413. doi: 10.1093/nar/gkt900 (PMC3919608; doi:10.1093/nar/gkt900)
Supplement: Supplementary Data [file supp_42_3_1393__index.html]

Comprehensive analysis of DNA polymerase III α subunits and their homologs in bacterial genomes — Comprehensive analysis of DNA polymerase III α subunits and their homologs in bacterial genomes — Supplementary Data 

# Comprehensive analysis of DNA polymerase III α subunits and their homologs in bacterial genomes

## Supplementary Data

files

**Files in this Data Supplement:**

- Supplementary Data - pdf file
- Supplementary Data - xls file
- Supplementary Data - xls file
