# Supplementary material for: Comprehensive analysis of DNA polymerase III α subunits and their homologs in bacterial genomes
Source: Nucleic Acids Res. 2013 Oct 6;42(3):1393–413. doi: 10.1093/nar/gkt900 (PMC3919608; doi:10.1093/nar/gkt900)
Supplement: Supplementary Data [file supp_gkt900_nar-02152-survey-d-2013-File017.pdf]

Comprehensive analysis of DNA polymerase III α subunits and their homologs in bacterial genomes

SUPPLEMENTARY FIGURES

Kęstutis Timinskas[1], Monika Balvočiūtė[1], Albertas Timinskas and Česlovas Venclovas*

Institute of Biotechnology, Vilnius University, Graičiūno 8, Vilnius LT-02241, Lithuania

[1]Joint first authors

*Corresponding author:

E-mail: venclovas@ibt.lt

Present address:

Monika Balvočiūtė, Institut für Mathematik und Informatik, Ernst Moritz Arndt Universität Greifswald, Germany

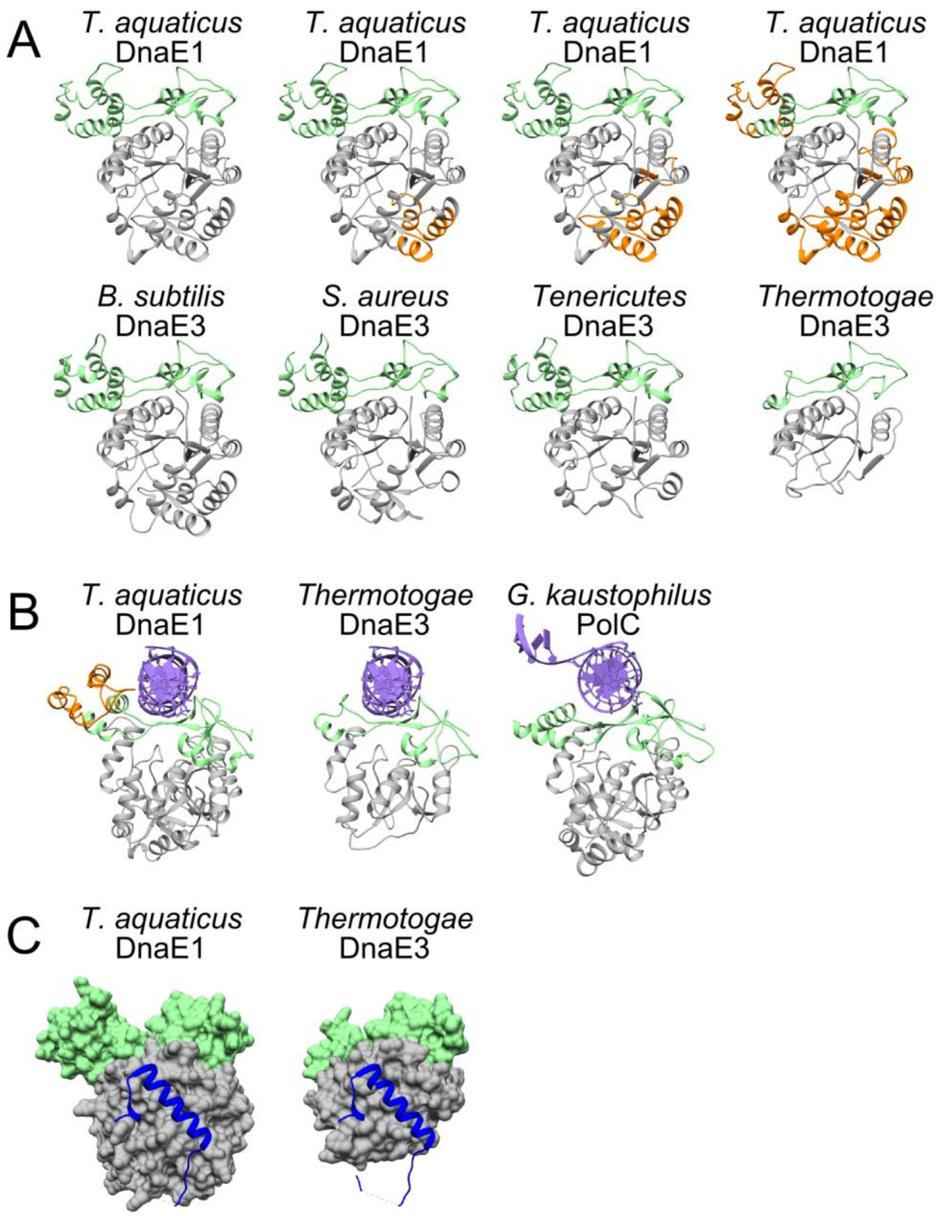

Supplementary Figure S1. Structural differences between *T. aquaticus* DnaE1 and various DnaE3 in PHP and Thumb domains. (A) Structures in ribbon representations colored according to structural domains and differences between groups. All structures are shown in the same orientation. Only the PHP domain (grey), the 'thumb' and a part of the 'palm' domains (light green) are shown. Structures in the first line (all depicting *Taq* DnaE1) are colored orange according to elements missing in structures in the second line. The structures used: crystal structure of *T. aquaticus* DnaE1 (PDB ID: 3E0D), models of *B. subtilis*, *Staphylococcus aureus* (both *Firmicutes*), *Mycoplasma capricolum* (*Tenericutes*), *Fervidobacter penivorans* (*Thermotogae*) DnaE3. (B) Contact site between the 'thumb' domain and DNA. For comparison, the same interaction in PolC is depicted (*G. kaustophilus*, PDB ID: 3F2B). The original DNA molecules are shown for DnaE1 and PolC. (C) C-terminal part of ε-subunit (PDB ID: 4GX9), as shown (in blue ribbon) superimposed onto structures of *T. aquaticus* DnaE1 and *Thermotogae* DnaE3 (solvent accessible surface representations). The missing structural elements in the PHP domain of Thermotogae DnaE3 constitute a large part of putative ε-subunit binding surface.

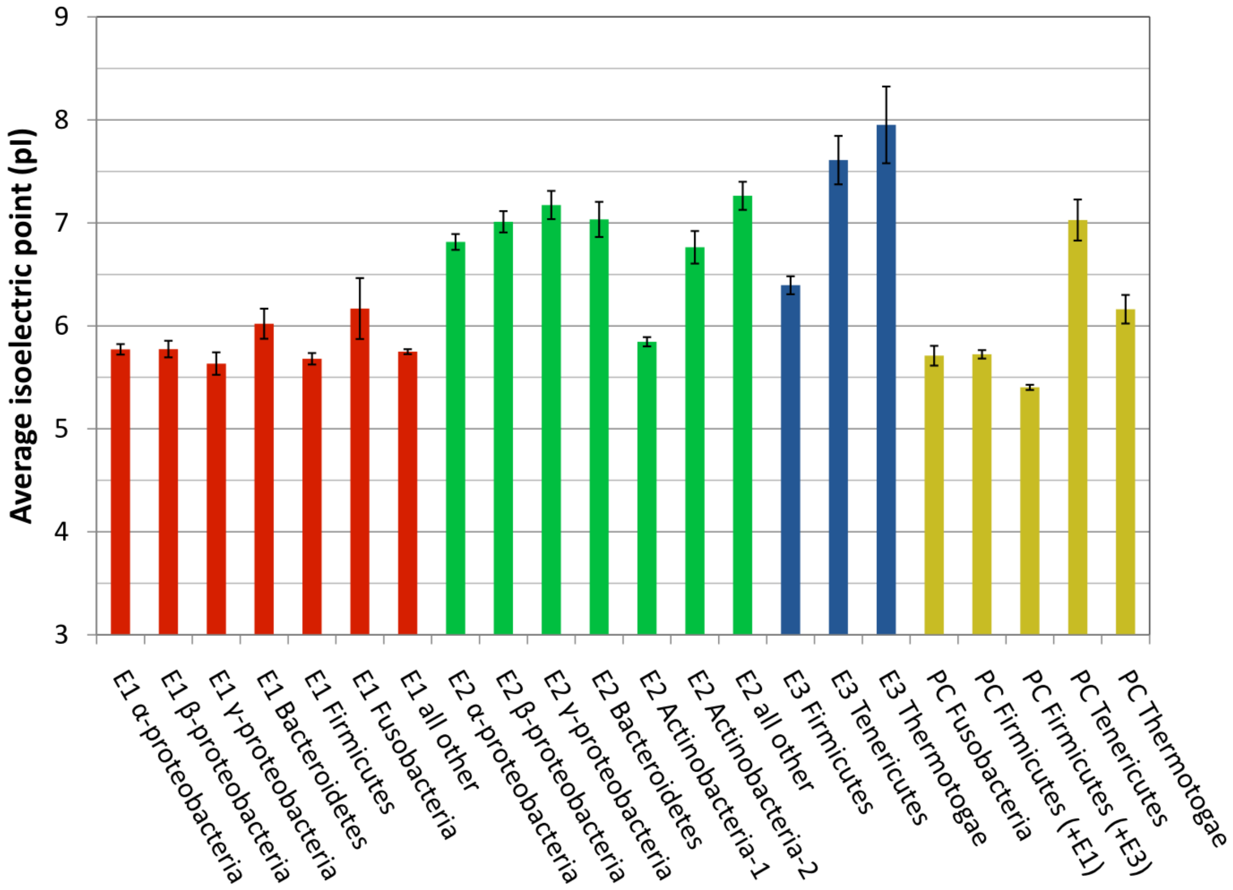

Supplementary Figure S2. Average isoelectric point (pI) values of polymerases in different subgroups. The isoelectric points are computed for the sequences corresponding to the universally conserved core (residues 6-889 of *E. coli* DnaE1). The subgroups are distinguished according to bacterial taxonomy. DnaE2 of *Actinobacteria* are split according to phylogenetic tree, PolC of *Firmicutes* are split according to accompanying polymerase (DnaE1 or DnaE3). See Figure S6 for more details on subgroups. Standard deviations of mean are indicated as error bars.

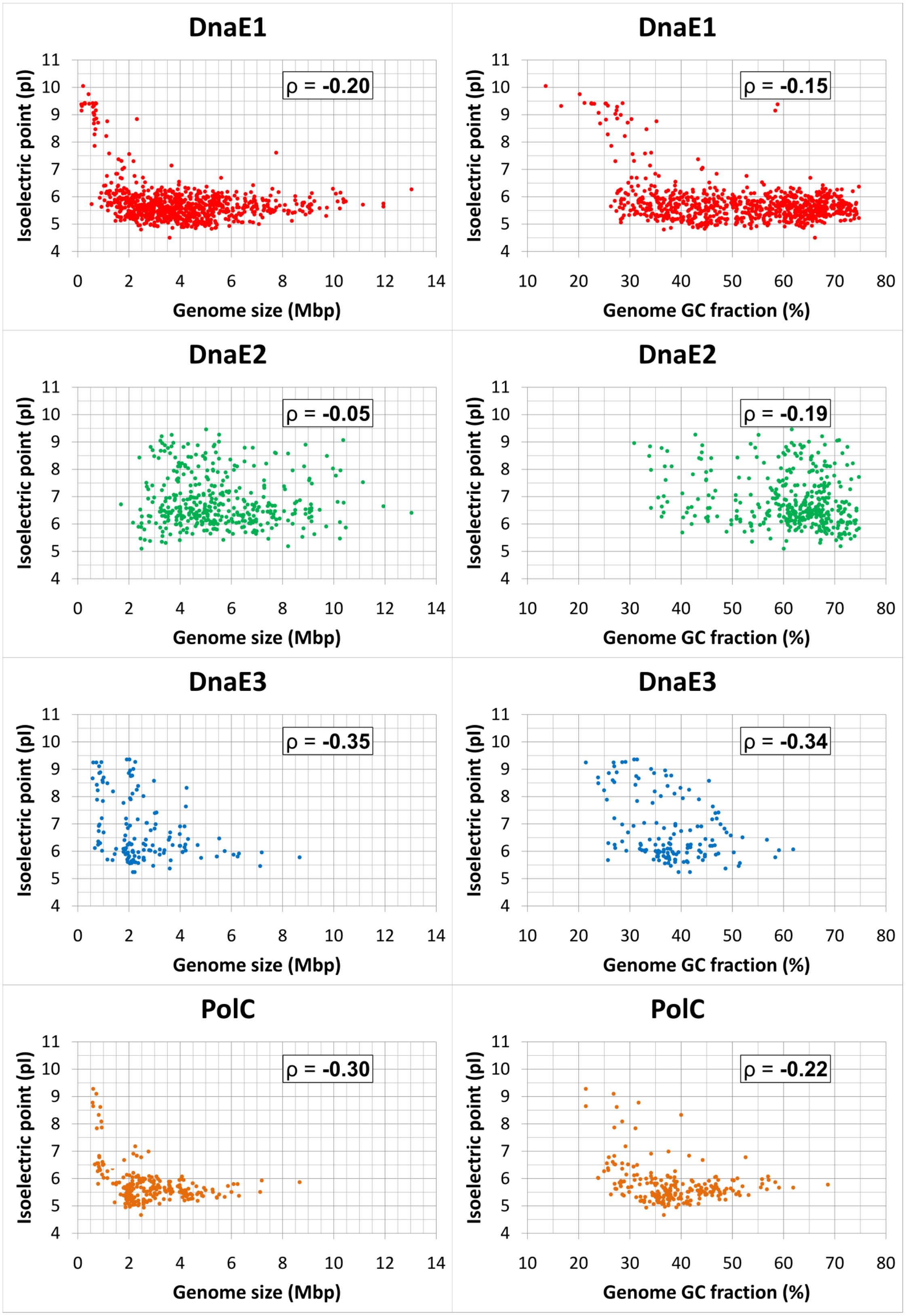

Supplementary Figure S3. Relationships between polymerase isoelectric point (pI) and genome size (left plots) or GC fraction (right plots) in all four polymerase groups. The isoelectric point was calculated for only the most conserved sequence region (PHP, Pol3 and $(HhH)_2$ domains; residues 6-889 in *E. coli* DnaE1). Spearman's rank correlation coefficient (ρ) is indicated in a box for each dataset.

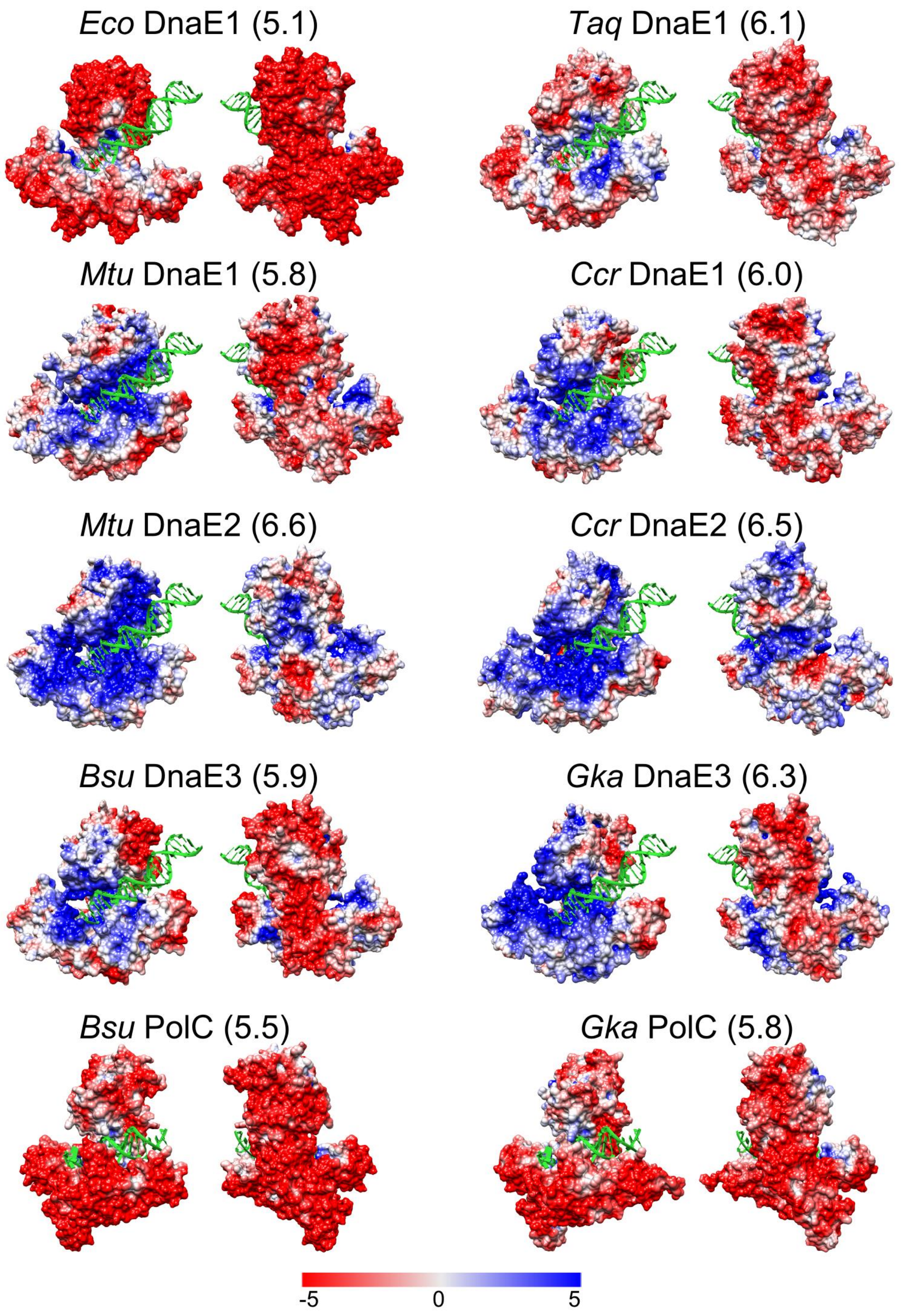

Supplementary Figure S4. Examples of surface electrostatics for members of different polymerase groups. The theoretical isoelectric points calculated for exact sequences of the shown structures are indicated in parentheses. All PDB structures and models were cut according to *E. coli* DnaE1 (PDB ID: 2HNH). All polymerases of selected bacteria are depicted for clear comparison: *E. coli* (*Eco*), *T. aquaticus* (*Taq*), *M. tuberculosis* (*Mtu*), *C. crescentus* (*Ccr*), *B. subtilis* (*Bsu*) and *G. kaustophilus* (*Gka*). Structures are shown in two orientations (same for all). The surfaces are colored according to assigned charge (red – negative, blue – positive, scale units – K_bT/e_c).

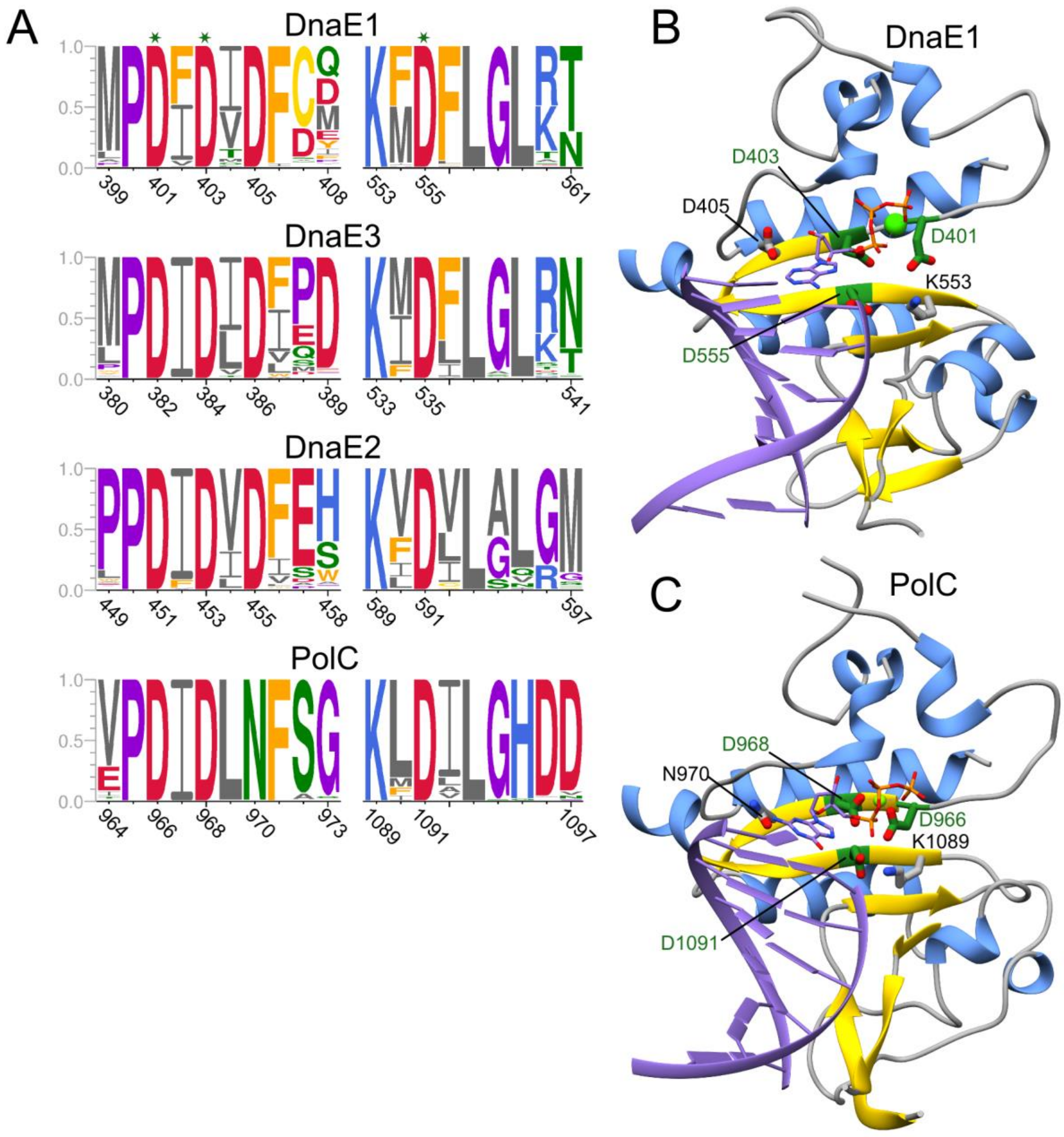

Supplementary Figure S5. Conservation of the active site region in different polymerase groups. (A) Conservation represented as sequence logos. Positions are numbered according to *E. coli* DnaE1, *M. tuberculosis* DnaE2, *B. subtilis* DnaE3 and PolC sequences respectively. Catalytic aspartates are indicated with green stars. Structures of active site regions ('palm' domains) complexed with DNA and the incoming nucleotide for (B) *T. aquaticus* DnaE1 (PDB ID 3E0D) and (C) *G. kaustophilus* PolC (PDB ID 3F2B). Catalytic aspartates are highlighted in green. Residue numbering is the same as in (A).

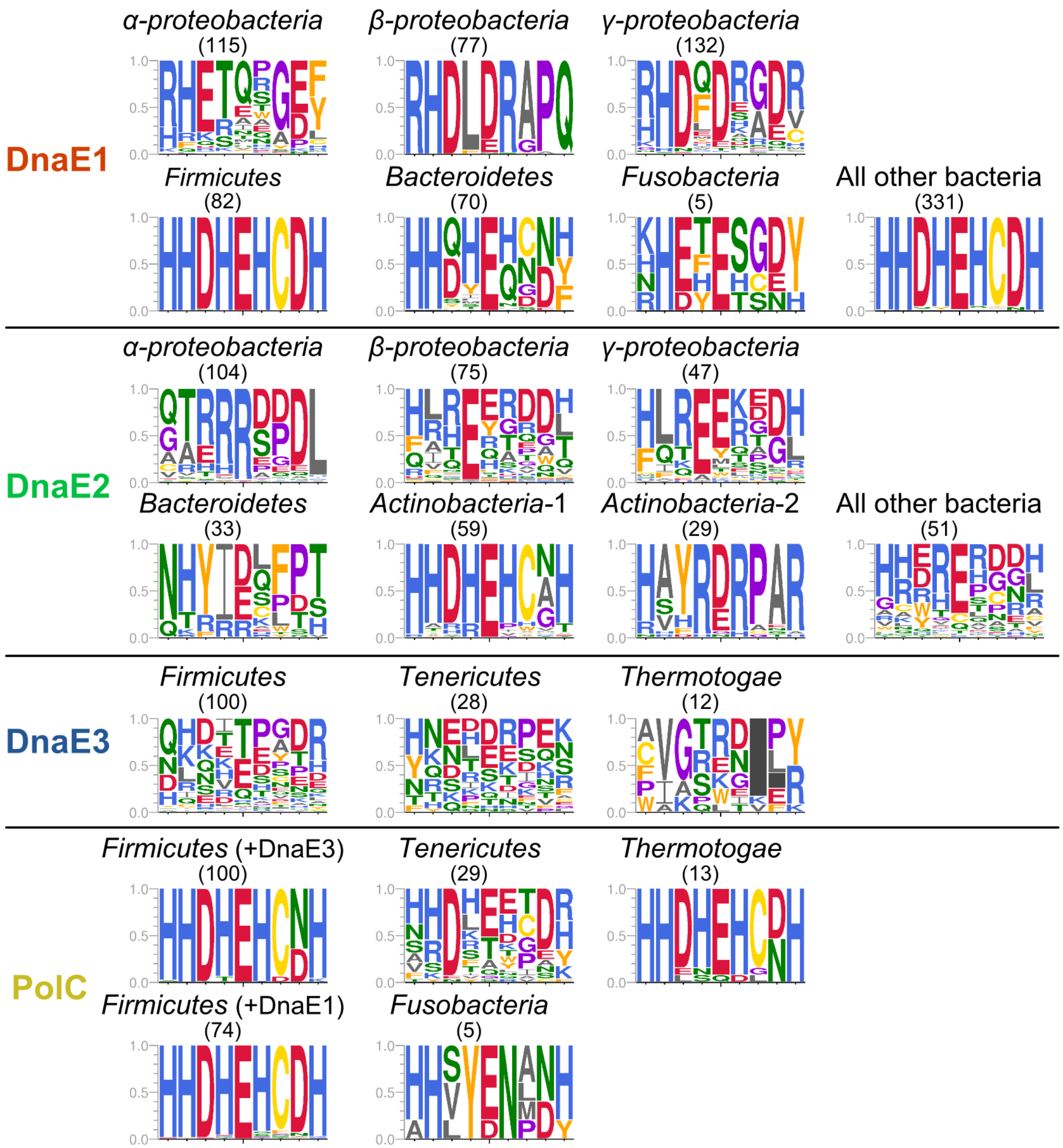

Supplementary Figure S6. Sequence logo representations of PHP metal-binding site in different polymerase groups, subdivided with respect to bacterial taxonomy. Corresponding positions (same for all subgroups) in *E. coli* DnaE1: 10, 12, 19, 44, 69, 83, 134, 201 and 203 (also refer to Figure 9 for corresponding positions in representatives of other polymerase groups). Numbers in parentheses denote the number of polymerases in the subgroup. ‘All other bacteria’ accounts for all polymerases of the group minus the ones already shown. DnaE2 of *Actinobacteria* are further split into two subgroups, according to the phylogenetic tree (100% bootstrap support for each of the two subgroups; *Actinobacteria*-1 is the group closer to the main proteobacterial cluster). Notable members of the *Actinobacteria*-1 subgroup are *Mycobacterium* and *Corynebacterium* genera; of *Actinobacteria*-2 – *Streptomyces* and *Arthrobacter* genera. PolC of *Firmicutes* are further split according to the accompanying polymerase found in the same genome (DnaE1 or DnaE3).

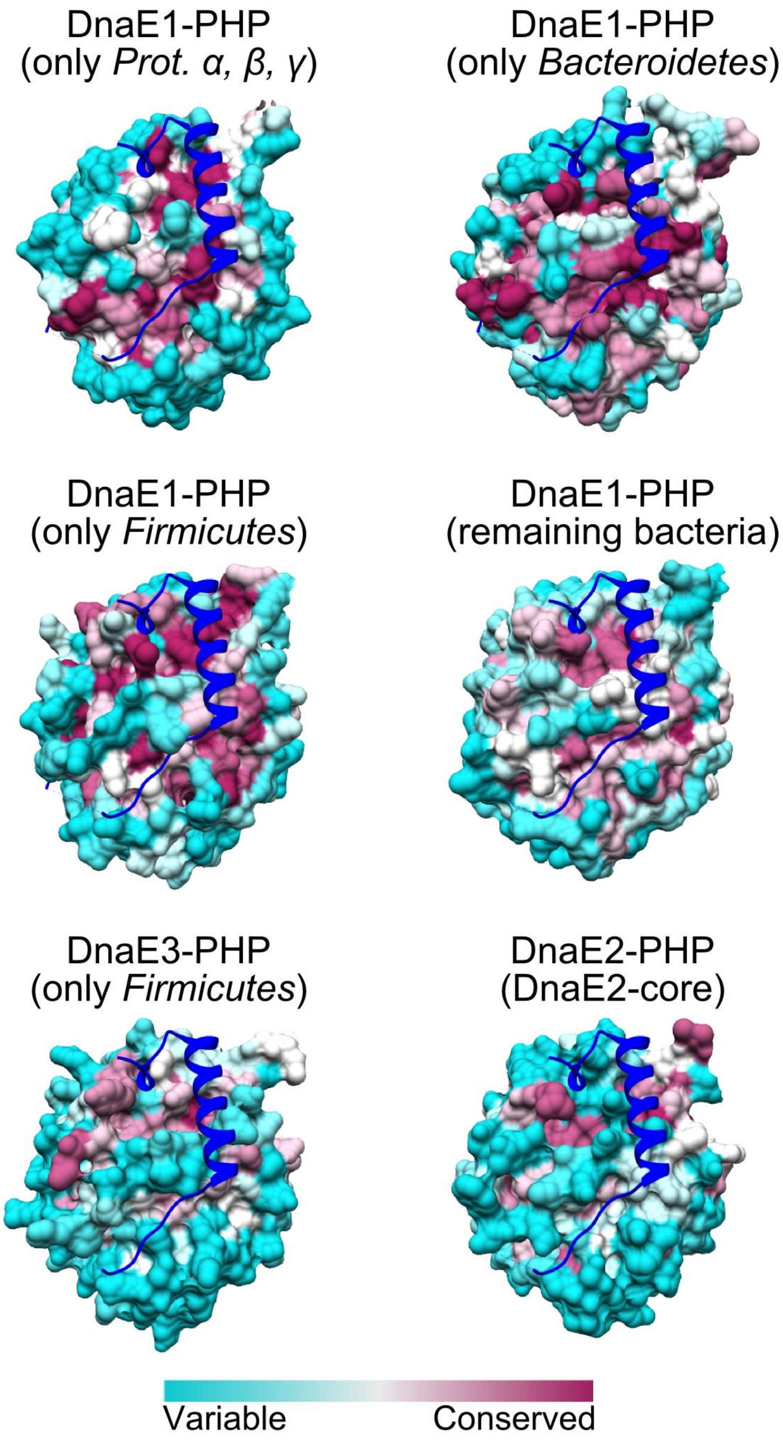

Supplementary Figure S7. Surface residue conservation at ε-subunit binding site in subgroups of DnaE. DnaE1 sequences are split into four subsets, according to bacterial taxonomy and conservation of PHP exonuclease active site: *Proteobacteria* (*α*-, *β*- and *γ*-) have low exonuclease active site conservation, *Bacteroidetes* have average conservation, *Firmicutes* and all other bacteria have very conserved PHP exonuclease active sites (Figure S6). For comparison, residue conservation for DnaE3 of *Firmicutes* is shown. Also, residue conservation for "core" group of DnaE2 (all DnaE2 with the identified C-terminal motif (Figure S9) are in this group) is shown (includes *α*-, *β*- and *γ-proteobacteria*, *Actinobacteria*-1 and some other bacteria (Figure 2)).

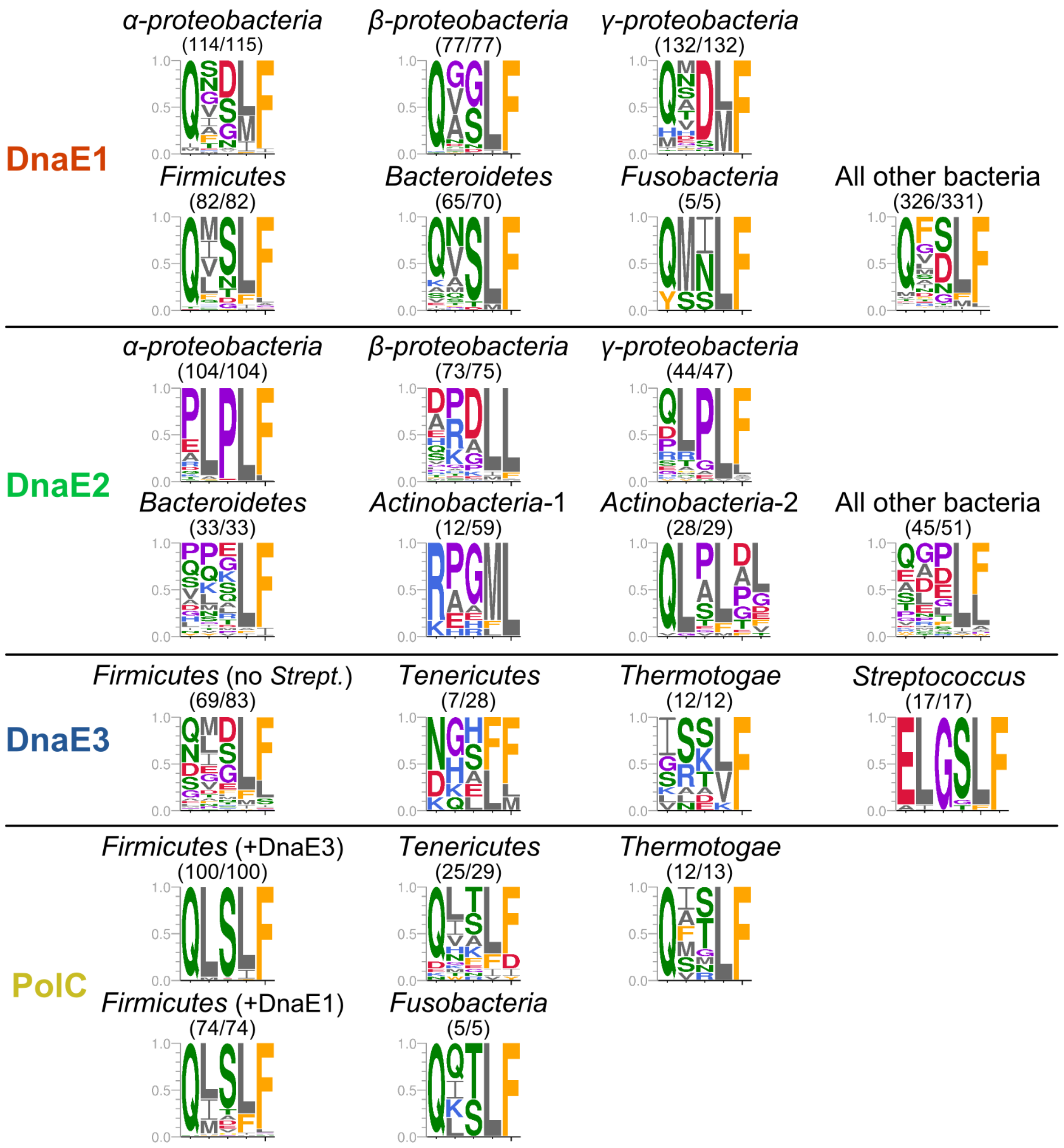

Supplementary Figure S8. Sequence logo representations of β-clamp binding motifs in different polymerase groups, subdivided with respect to bacterial taxonomy. Sequence counts in which the motif was identified and the total number of sequences belonging to the subgroup are indicated in parentheses. The corresponding motif in *E. coli* DnaE1 is in positions 920-924. Subgroups are similar to the ones shown in Figure S6. The only difference is that genus *Streptococcus* is split from *Firmicutes* DnaE3. These DnaE3 in bacteria of genus *Streptococcus* for the clamp-binding may potentially use a distinct hexameric motif (ELGSLF), in which a single residue in the position 3 is replaced with two small residues (GS). In at least half of DnaE2 of the second actinobacterial group the β-clamp binding motif could possibly be extended to a hexameric motif including an additional Leu.

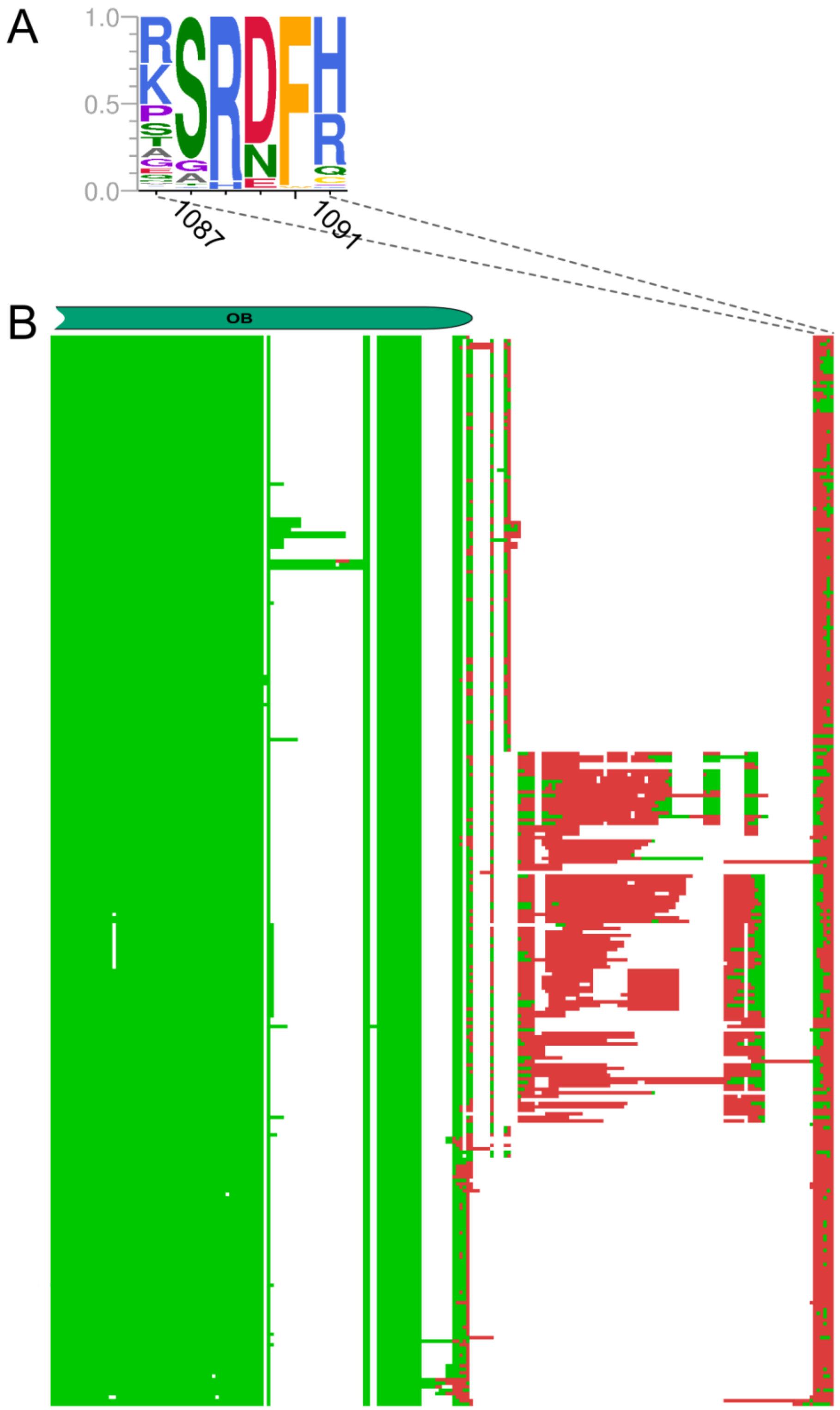

Supplementary Figure S9. A highly conserved C-terminal motif identified in part of DnaE2 sequences. (A) A logo representation of the motif (*M. tuberculosis* DnaE2 residues 1087-1091). (B) Disordered region (red) at the C-terminal part of DnaE2 sequences, represented as a multiple sequence alignment of all DnaE2 that contain the motif (306 sequences). Only residues corresponding to *M. tuberculosis* DnaE2 residues 1003-1091 are depicted.

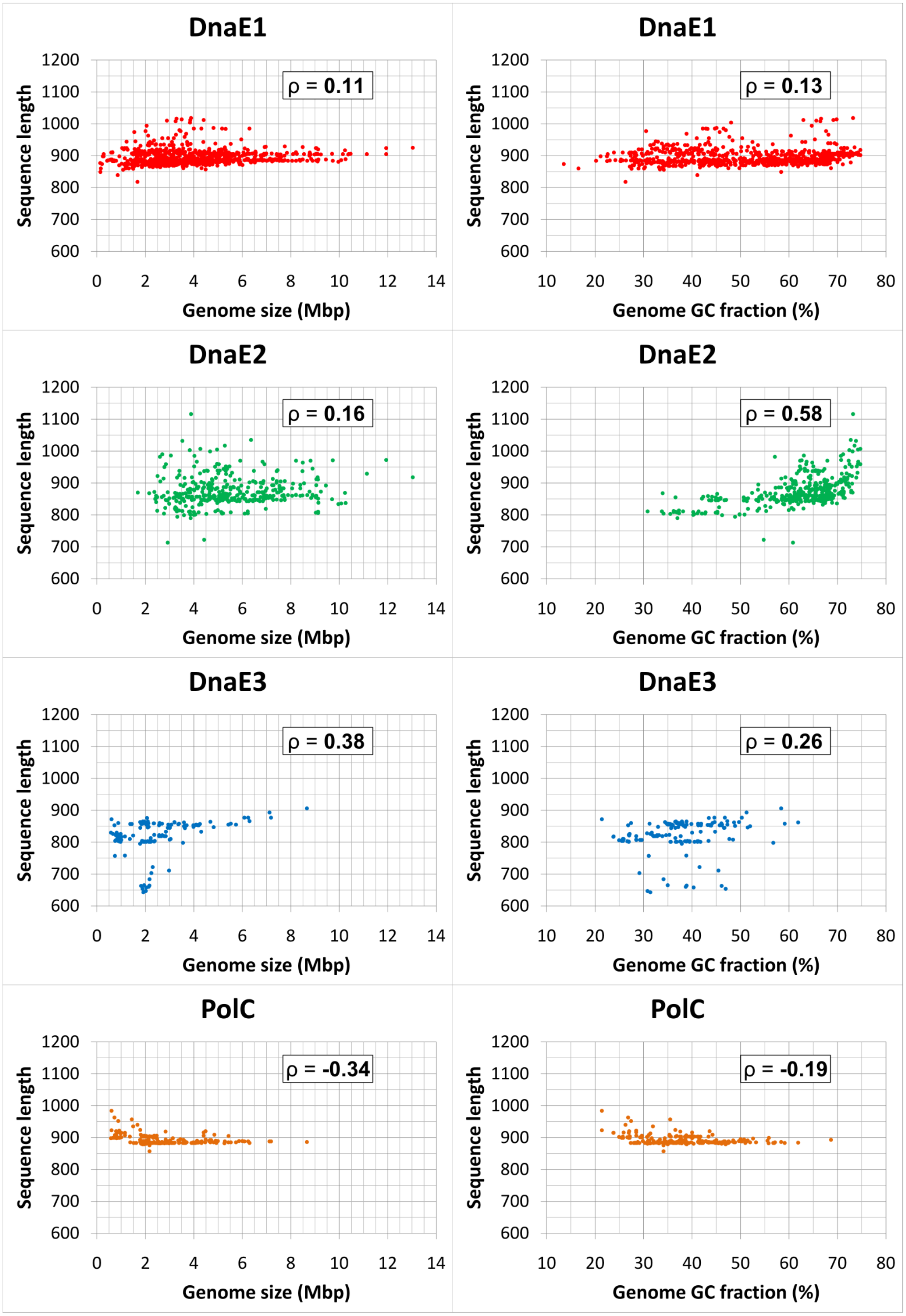

Supplementary Figure S10. Relationships between polymerase sequence length and genome size (left plots) or GC fraction (right plots) in all four polymerase groups. The sequence length corresponds to only the most conserved sequence region (PHP, Pol3 and $(HhH)_2$ domains; residues 6-889 in *E. coli* DnaE1). Spearman's rank correlation coefficient (ρ) is indicated in a box for each dataset.
